# All Hierarchical Core–Shell Heterostructures as Novel Binder‐Free Electrode Materials for Ultrahigh‐Energy‐Density Wearable Asymmetric Supercapacitors

**DOI:** 10.1002/advs.201801379

**Published:** 2018-11-12

**Authors:** Qiulong Li, Qichong Zhang, Juan Sun, Chenglong Liu, Jiabin Guo, Bing He, Zhenyu Zhou, Ping Man, Chaowei Li, Liyan Xie, Yagang Yao

**Affiliations:** ^1^ Division of Advanced Nanomaterials Key Laboratory of Nanodevices and Applications Joint Key Laboratory of Functional Nanomaterials and Devices CAS Center for Excellence in Nanoscience Suzhou Institute of Nanotech and Nanobionics Chinese Academy of Sciences Suzhou 215123 P. R. China; ^2^ National Laboratory of Solid State Microstructures College of Engineering and Applied Sciences, and Collaborative Innovation Center of Advanced Microstructures Nanjing University Nanjing 210093 China; ^3^ Division of Nanomaterials Suzhou Institute of Nano‐Tech and Nano‐Bionics, Nanchang Chinese Academy of Sciences Nanchang 330200 China

**Keywords:** CoNiO_2_@Ni(OH)_2_, core–shell nanostructures, fiber‐shaped asymmetric supercapacitors, TiN@VN, wearable electronics

## Abstract

High‐performance fiber‐shaped energy‐storage devices are indispensable for the development of portable and wearable electronics. Composite pseudocapacitance materials with hierarchical core–shell heterostructures hold great potential for the fabrication of high‐performance asymmetric supercapacitors (ASCs). However, few reports concerning the assembly of fiber‐shaped ASCs (FASCs) using cathode/anode materials with all hierarchical core–shell heterostructures are available. Here, cobalt‐nickel‐oxide@nickel hydroxide nanowire arrays (NWAs) and titanium nitride@vanadium nitride NWAs are constructed skillfully with all hierarchical core–shell heterostructures directly grown on carbon nanotube fibers and are shown to exhibit ultrahigh capacity and specific capacitance, respectively. The specific features and outstanding electrochemical performances of the electrode materials are exploited to fabricate an FASC device with a maximum working voltage of 1.6 V, and this device exhibits a high specific capacitance of 109.4 F cm^−3^ (328.3 mF cm^−2^) and excellent energy density of 36.0 mWh cm^−3^ (108.1 µWh cm^−2^). This work therefore provides a strategy for constructing all hierarchical core–shell heterostructured cathode and anode materials with ultrahigh capacity for the fabrication of next‐generation wearable energy‐storage devices.

## 1 Introduction

The rapidly growing demand for the miniaturization of wearable and portable electronics necessitates the development of energy storage devices with high energy density.^1^, ^2^, ^3^, ^4^, ^5^, ^6^, ^7^, ^8^, ^9^, ^10^, ^11^, ^12^, ^13^, ^14^, ^15^, ^16^, ^17^ Supercapacitors (SCs), especially fiber‐shaped supercapacitors (FSCs) with their tiny volume, remarkable flexibility, and excellent stitchability as well as fast charge–discharge capability, a high power density, and a long cycle life, are promising energy storage and supply devices.^4^, ^8^, ^11^, ^18^, ^19^, ^20^, ^21^, ^22^, ^23^, ^24^, ^25^, ^26^, ^27^ However, their low energy density has severely restricted their practical application in high‐energy‐density wearable devices.^25^, ^28^, ^29^, ^30^, ^31^, ^32^ Additionally, low specific capacitance of fiber‐shaped asymmetric supercapacitors (FASCs) has limited further improvements in the energy density of these devices.^21^, ^29^, ^33^, ^34^ These restrictions mainly originate from the characteristics of the electrode materials. Thus, it is imperative to develop highly capacitive cathode and anode materials for use in high‐performance FASCs.

Among the various pseudocapacitive cathode materials, bimetallic transition‐metal oxides are the most promising candidates owing to their remarkable electrochemical capability, high electronic conductivity, low cost, and environmental friendliness.^1^, ^9^, ^19^, ^23^, ^28^, ^35^, ^36^, ^37^ However, further enhancing the capacity of single‐component bimetallic oxides remains challenging.^1^, ^35^, ^36^ An effective strategy for further improving the capacity of the cathode materials is to fabricate hierarchical core–shell heterostructures consisting of a core material with high electrical conductivity and a shell layer based on the well‐known pseudocapacitive transition‐metal oxides/hydroxides.^1^, ^11^, ^36^, ^38^, ^39^ Therefore, in this work, cobalt‐nickel‐oxide (CoNiO_2_) nanowire arrays (NWAs) were selected for their high electrical conductivity and a certain amount of capacity.^23^ These outstanding features make CoNiO_2_ NWAs an excellent secondary substrate for growing other active materials with a nanosheet (NS) structure. Hence, a facile and cost‐effective method was used to directly grow 3D well‐aligned hierarchical CoNiO_2_ NWAs@nickel hydroxide (Ni(OH)_2_) NSs core–shell heterostructures on a carbon nanotube fiber (CNTF) as a novel binder‐free positive electrode. The intriguing structural features of the CoNiO_2_ NWAs@Ni(OH)_2_ NSs/CNTF electrode afforded an ultrahigh capacity of 0.674 mAh cm^−2^, which is significantly higher than the values reported for other fibrous cathode materials.^1^, ^4^, ^6^, ^8^, ^20^, ^21^, ^22^, ^34^, ^40^, ^41^

Anode materials with high electrical conductivity and specific capacitance also play a crucial role in the fabrication of high‐energy‐density FASCs.^33^, ^42^ Metallic nitrides are emerging as novel and promising electrode materials for high‐performance FASCs owing to their outstanding electrical conductivity and relatively high capacitance.^42^, ^43^, ^44^, ^45^, ^46^ In particular, titanium nitride (TiN) holds great potential as an electrode material because of its excellent electrical conductivity and mechanical stability.^42^, ^44^, ^45^, ^47^, ^48^, ^49^ The excellent characteristics of TiN also make it an attractive secondary substrate for growing other active materials with a NS structure. Vanadium nitride (VN) has also attracted considerable attention due to its good electrical conductivity, large work window, fast reversible Faradaic redox reactions, and high specific capacitance.^33^, ^42^, ^50^, ^51^, ^52^ Therefore, a highly efficient and cost‐effective method was applied to directly grow 3D well‐aligned hierarchical TiN NWAs@VN NSs core–shell heterostructures on a CNTF as a novel binder‐free negative electrode. Due to the unique core–shell structure, the TiN NWAs@VN NSs/CNTF electrode exhibit a very high specific capacitance of 1255.2 mF cm^−2^ (418.4 F cm^−3^), which is much higher than previously reported fibrous negative electrode.^24^, ^33^

The cathode and anode materials based on core–shell heterostructures (CoNiO_2_ NWAs@Ni(OH)_2_ NSs/CNTF and TiN NWAs@VN NSs/CNTF) were exploited for the rational design and construction of a high‐performance FASC with a maximum working voltage of 1.6 V. As a new type of wearable energy‐storage device, the assembled FASC exhibited very high specific capacitance and energy density. Furthermore, the fabricated FASC also displayed very flexible and stable performance, which may permit its commercialization as an energy‐storage device for automotive and wind turbine applications.

## 2 Results and Discussion

**Figure 1:**
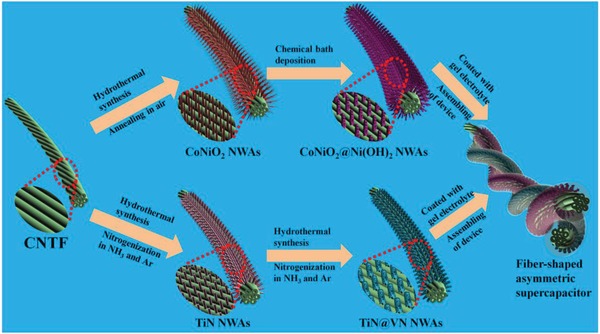
Schematic diagram of the fabrication of the cathode and anode materials and the FASC device.

**Figure** **1** schematically depicts the synthetic route used to prepare the hierarchical CoNiO_2_ NWAs@Ni(OH)_2_ NSs/CNTF and TiN NWAs@VN NSs/CNTF core–shell heterostructure electrodes and the FASC device. To obtain the positive electrode, CoNiO_2_ NWAs were grown on a CNTF via a two‐step process consisting of hydrothermal synthesis followed by annealing in air. Ni(OH)_2_ NSs were then deposited on the CoNiO_2_ NWAs by chemical bath deposition. To obtain the negative electrode, TiN NWAs were first grown on a CNTF via a two‐step process consisting of hydrothermal synthesis followed by nitrogenization treatment in ammonia/argon. Subsequently, VN NSs were grown on the TiN NWAs using the same two processes, i.e., hydrothermal synthesis followed by nitrogenization treatment in ammonia/argon. Finally, the FASC device was assembled by twisting the two electrodes together with a gel electrolyte coating. CoNiO_2_ and TiN possess many promising properties such as high electrochemical activity, electrical conductivity, and chemical stability.^23^, ^44^, ^45^ In this study, the CoNiO_2_ and TiN NWAs grown on the CNTFs not only act as electrically conductive and stable supports for the growth of the nanostructured metal hydroxide and metal nitride that serve as the cathode and anode, respectively, but also promote ultrafast charge transfer and ion diffusion, which significantly improve the capacitive performance.

**Figure 2:**
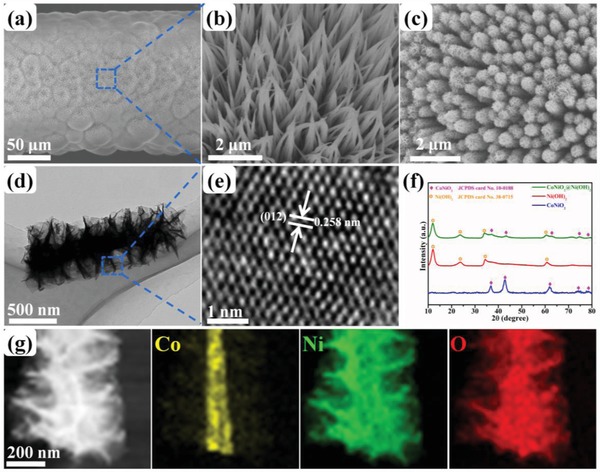
a,b) SEM images of CoNiO_2_ NWAs/CNTF at different magnifications. c) SEM image of CoNiO_2_@Ni(OH)_2_ NWAs/CNTF. d) Low‐magnification TEM image of single CoNiO_2_@Ni(OH)_2_ NWAs. e) High‐magnification TEM image of the blue rectangle in panel (d). f) XRD patterns of the CoNiO_2_@Ni(OH)_2_ NWAs, pure CoNiO_2_ NWAs, and Ni(OH)_2_ nanosheets. g) TEM image of the CoNiO_2_@Ni(OH)_2_ NWAs and the corresponding energy‐dispersive spectroscopy element mapping images.

A CNTF was used for current collector that was prepared by twisting a carbon nanotube trip via a fast and scale process (Figure S1, Supporting Information). Figure S2 in the Supporting Information displays the scanning electron microscope (SEM) images of the bare CNTF with different magnification. It can be clearly seen that the bare CNTF shows a uniform diameter with 110 µm (Figure S2a, Supporting Information) and the aligned CNT in the CNTF is also displayed (Figure S2b, Supporting Information). **Figure** **2**a clearly shows that the hydrothermal synthesis and subsequent high‐temperature calcination resulted in the uniform growth of the CoNiO_2_ NWAs on the CNTF surface. Figure 2b shows that the CoNiO_2_ NWAs were evenly distributed and highly aligned on the surface of the CNTF. The transmission electron microscope (TEM) results presented in Figure S3 in the Supporting Information further confirm the microstructure, lattice distance, and elemental distribution of the single CoNiO_2_ nanowire. Figure 2c demonstrates that the chemical bath deposition process afforded homogeneous loading of the ultrathin Ni(OH)_2_ NSs on the surfaces of the CoNiO_2_ NWAs to form unique and 3D hierarchical core–shell heterostructures. Figure S4 in the Supporting Information shows the microstructure of the Ni(OH)_2_ NSs directly grown on a CNTF. Figure S5 in the Supporting Information compares the stress–strain curves of pristine CNTF, CoNiO_2_ NWAs/CNTF, and CoNiO_2_ NWAs@Ni(OH)_2_ NSs/CNTF under the same experimental conditions. The CoNiO_2_ NWAs@Ni(OH)_2_ NSs/CNTF possesses an excellent tensile strength of 349 MPa and a tensile strain of 1.45%, which is of great importance for its weavability. Furthermore, we made a longer CoNiO_2_ NWAs@Ni(OH)_2_ NSs/CNTF electrode and wove it into a piece of white cloth to better display its weavability, as shown in Figure S6 in the Supporting Information. The results exhibit that the CoNiO_2_ NWAs@Ni(OH)_2_ NSs/CNTF electrode shows a prominent weavability. TEM was also used to observe the morphology of the core–shell heterostructure in more detail, as shown in Figure 2d,e. It can be clearly seen that the CoNiO_2_ NWAs@Ni(OH)_2_ NSs possessed a typical core–shell heterostructure. Furthermore, the high‐magnification image reveals the presence of lattice fringes with a spacing of 0.258 nm, corresponding to the (012) plane of α‐Ni(OH)_2_. The chemical compositions of the core and shell regions were also examined by X‐ray diffraction (XRD) measurements as shown in Figure 2f. The diffraction peaks at 36.8°, 42.8°, 61.8°, 74.0°, and 78.0° were indexed to the (111), (200), (220), (311), and (222) phases of CoNiO_2_ (Joint Committee on Powder Diffraction Standards (JCPDS) Card No. 10‐0188), respectively, whereas those centered at 11.3°, 22.9°, 34.0°, and 60.4° were indexed to the (003), (006), (012), and (110) phases of α‐Ni(OH)_2_ (JCPDS Card No. 38‐0715), respectively. The XRD pattern of the CoNiO_2_ NWAs@Ni(OH)_2_ NSs contained the major diffraction peaks for both CoNiO_2_ and Ni(OH)_2_ without any additional peaks, indicating that the fabricated core–shell heterostructures consisted solely of CoNiO_2_ and α‐Ni(OH)_2_. Figure 2g clearly shows that the CoNiO_2_ NWAs@Ni(OH)_2_ NSs possessed a hierarchical core–shell structure, in which the Co was entirely derived from CoNiO_2_ and the Ni and O were derived from both CoNiO_2_ and Ni(OH)_2_. The chemical compositions and valence states of the CoNiO_2_ NWAs@Ni(OH)_2_ NSs were further investigated using X‐ray photoelectron spectroscopy (XPS) (Figure S7, Supporting Information). These data further confirmed the hierarchical CoNiO_2_ core/Ni(OH)_2_ shell structure and composition.

**Figure 3:**
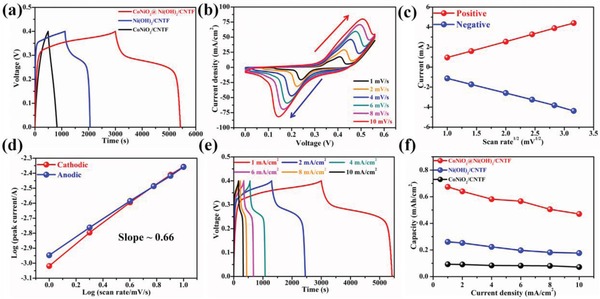
a) GCD curves for the CoNiO_2_@Ni(OH)_2_ NWAs/CNTF, CoNiO_2_ NWAs/CNTF, and Ni(OH)_2_/CNTF electrodes at a current density of 1 mA cm^−2^. b) CV curves for the CoNiO_2_@Ni(OH)_2_ NWAs/CNTF electrode at various scan rates. c) Plots of the cathodic and anodic peak currents versus the square root of the scan rate. d) Linear fitting of the peak current versus scan rate for the cathodic and anodic peaks of the CV curves shown in (b). e) GCD curves for the CoNiO_2_@Ni(OH)_2_ NWAs/CNTF electrode at various current densities. f) Capacity of the three electrodes as a function of current density.

The electrochemical performances of pristine CNTF and as‐fabricated fibrous electrodes were evaluated using a three‐electrode measuring system in 3 m potassium hydroxide (KOH) aqueous electrolyte solution. Figure S8 in the Supporting Information presents the electrochemical performance of bare CNTF used as positive electrode. The contribution of the electrochemical performance of the bare CNTF is completely negligible for our as‐fabricated CoNiO_2_ NWAs@Ni(OH)_2_ NSs/CNTF electrode. **Figure** **3** shows the electrochemical performance characteristics of the as‐fabricated positive electrode. Comparison of the galvanostatic charge/discharge (GCD) curves recorded at 1 mA cm^−2^ revealed a significantly higher capacity for the CoNiO_2_ NWAs@Ni(OH)_2_ NSs/CNTF electrode than for the CoNiO_2_ NWAs/CNTF and Ni(OH)_2_ NSs/CNTF electrodes (Figure 3a). The cyclic voltammetry (CV) and GCD profiles of the CoNiO_2_ NWAs/CNTF and Ni(OH)_2_ NSs/CNTF electrodes are shown in Figures S9 and S10 in the Supporting Information, respectively, for comparison. Interestingly, the CV curves of the CoNiO_2_ NWAs@Ni(OH)_2_ NSs/CNTF electrode enclosed much larger areas than those of the CoNiO_2_ NWAs/CNTF and Ni(OH)_2_ NSs/CNTF electrodes at various scan rates, further demonstrating the superior electrochemical performance of the CoNiO_2_ NWAs@Ni(OH)_2_ NSs/CNTF electrode. Therefore, a high charge can be stored in the CoNiO_2_ NWAs@Ni(OH)_2_ NSs/CNTF electrode. The superior electrochemical performance of the CoNiO_2_ NWAs@Ni(OH)_2_ NSs/CNTF electrode was ascribed to the high electrical conductivity of the CoNiO_2_ core and the synergistic effects of the well‐aligned CoNiO_2_ NWAs and Ni(OH)_2_ NSs. Electrochemical impedance spectroscopy (EIS) was also applied to evaluate the electrochemical behavior of the three cathodes and the corresponding Nyquist plots are presented in Figure S11 in the Supporting Information. Furthermore, the compacted electrical resistance of the three metallic compound powders was measured by using four‐probe for comparison, as shown in Figure S12 in the Supporting Information. These results further demonstrate the superiority of the CoNiO_2_ NWAs as the core electrode material. As the scan rate was increased from 1 to 10 mV s^−1^, the overall shapes of the CV curves of the CoNiO_2_ NWAs@Ni(OH)_2_ NSs/CNTF electrode exhibited little variation (Figure 3b). However, obvious shifts in the positions of the anodic and cathodic peaks toward more positive and more negative potentials, respectively, were observed as the scan rate was increased from 1 to 10 mV s^−1^, which were mainly the result of the increased resistance and electric polarization of the cathode material. Additionally, the linear relationship between the square root of the scan rate and the redox peak current at various scan rates is shown in Figure 3c, demonstrating that the redox reaction of the electrode materials was a quasi‐reversible and diffusion‐controlled process.^36^ Figure 3d shows the relationships between log(peak current, *i*) and log(scan rate, υ) from 1 to 10 mV s^−1^ for both the cathodic and anodic peaks. Assuming that the tested current complies with a power‐law relationship with the scan rate, according to Equation (1), 53, 54

(1) $$i=\alpha \upsilon^{\beta }$$

where α and β are adjustable parameters; β can be determined from the slope of the plot of log(*i*) versus log(υ). On the basis of the approximations, β = 0.5 indicates a current controlled by semi‐infinite linear diffusion, whereas β = 1 indicates a surface‐controlled current. From Figure 3d, the slopes of the lines were calculated to be approximately 0.66, indicating that the redox reactions of CoNiO_2_ NWAs@Ni(OH)_2_ NSs exhibited both surface‐controlled and diffusion‐controlled kinetics. Furthermore, the same results were also observed for Ni(OH)_2_ NSs/CNTF (Figure S13, Supporting Information). The GCD curves of the CoNiO_2_ NWAs@Ni(OH)_2_ NSs/CNTF electrode in the voltage range from 0 to 0.4 V at various current densities are presented in Figure 3e. It can be clearly seen that the voltage plateaus observed in the GCD curves matched well with the CV tests. In addition, the charging and discharging times at each current density were almost identical, demonstrating the outstanding reversibility of the redox reactions. Figure 3f shows the capacity of the three electrodes as a function of the current density, which were calculated from the GCD curves. The CoNiO_2_ NWAs@Ni(OH)_2_ NSs/CNTF electrode exhibited the highest capacity of 0.674 mAh cm^−2^ at a current density of 1 mA cm^−2^, and an impressively high capacity of 0.470 mAh cm^−2^ was maintained even at the high current density of 10 mA cm^−2^. Table S1 in the Supporting Information summarizes the comparison of the capacity and rate capability of our positive electrode with previously reported works, indicating more excellent electrochemical performance of the CoNiO_2_ NWAs/Ni(OH)_2_ NSs/CNTF. Therefore, the CoNiO_2_ NWAs/Ni(OH)_2_ NSs with a core–shell structure exhibited high energy‐storage performance and are a promising battery‐type electrode active material for the construction of high‐performance FASCs.

**Figure 4:**
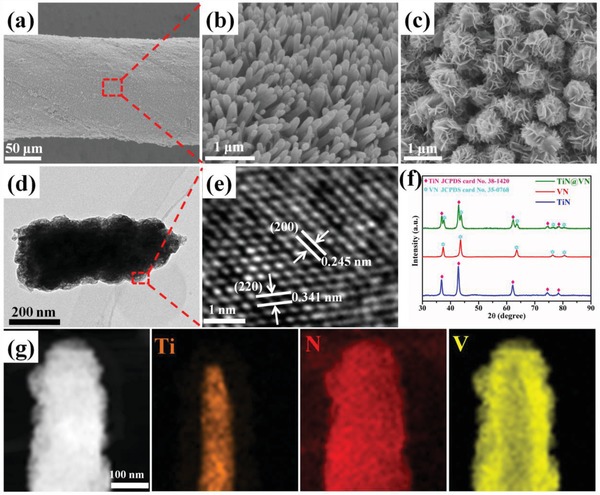
a,b) SEM images of TiN NWAs/CNTF at different magnifications. c) SEM image of TiN@VN NWAs/CNTF. d) Low‐magnification TEM image of the TiN NWA@VN NSs. e) High‐magnification TEM image of the red rectangle in panel (d). f) XRD patterns of the TiN@VN NWAs, pure VN NSs, and TiN NWAs. g) TEM image of the TiN NWA@VN NSs and the corresponding energy‐dispersive spectroscopy element mapping images.

The TiN NWAs were uniformly grown on the CNTF surface by hydrothermal synthesis and subsequent high‐temperature nitrogenization (**Figure** **4**a). The as‐fabricated TiN possessed a nanowire structure with a high degree of alignment (Figure 4b). The TEM results for a single TiN nanowire shown in Figure S14 in the Supporting Information further confirm the microstructure, lattice distance, and elemental distribution. As shown in Figure 4c, the VN NSs were homogeneously anchored on the surfaces of the TiN NWAs to form unique and 3D hierarchical core–shell heterostructures. The VN NSs were also uniformly grown directly on the CNTFs for comparison (Figure S15, Supporting Information). Figure S16 in the Supporting Information shows the tensile property of pristine CNTF, TiN NWAs/CNTF, and TiN NWAs@VN NSs/CNTF, demonstrating that the TiN NWAs@VN NSs/CNTF have a remarkable tensile strength of 381 MPa and a tensile strain of 1.65%, which is of great importance for its weavability. Additionally, the TiN NWAs@VN NSs/CNTF electrode also presents an outstanding weavability (Figure S17, Supporting Information). The TEM results also confirmed the TiN NWAs@VN NSs core–shell heterostructures (Figure 4d), and the high‐magnification image revealed the presence of lattice fringes with spacing of 0.245 and 0.341 nm, corresponding to the (200) and (220) plane of the VN shell, respectively (Figure 4e). Figure 4f shows the XRD patterns of the as‐fabricated samples, which confirmed the chemical composition of the core and shell. The diffraction peaks at 36.6°, 42.6°, 61.8°, 74.1°, and 77.9° were indexed to the (111), (200), (220), (311), and (222) phases of TiN (JCPDS Card No. 38‐1420), respectively, whereas those at 37.6°, 43.7°, 63.5°, 76.2°, and 80.3° were indexed to the (111), (200), (220), (311), and (222) phases of VN (JCPDS Card No. 35‐0768), respectively. These results confirm that the core and shell consisted of TiN and VN, respectively. The XRD patterns of the TiN NWAs@VN NSs contained all of the diffraction peaks for both TiN and VN without any additional peaks, indicating that the fabricated core–shell heterostructures consisted solely of TiN and VN. Furthermore, the hierarchical core–shell structure of single TiN NWAs@VN NSs was clearly observed by TEM, in which the Ti and V were completely derived from the TiN core and VN shell, respectively (Figure 4g). The chemical compositions and valence states of the TiN NWAs@VN NSs were investigated using XPS (Figure S18, Supporting Information). These data further confirmed the hierarchical TiN core @VN shell structure and composition.

**Figure 5:**
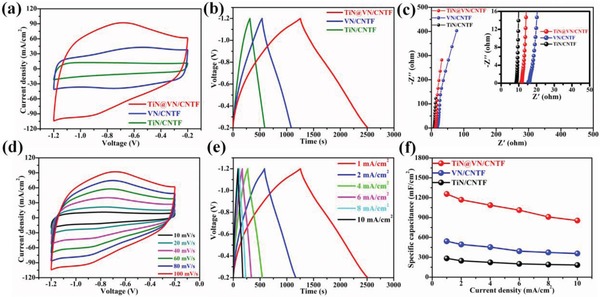
a) CV curves for the three electrodes at a scan rate of 100 mV s^−1^. b) GCD curves for the three electrodes at a current density of 1 mA cm^−2^. c) Nyquist plots for the three electrodes (the inset shows an enlarged section of the Nyquist plots). d) CV curves of the TiN@VN NWAs/CNTF electrode at various scan rates. e) GCD curves of the TiN@VN NWAs/CNTF electrode at various current densities. f) Specific capacitances of the three electrodes calculated from the GCD curves as a function of current density.

**Figure** **5** shows the electrochemical performance characteristics of the as‐fabricated negative electrode in 3 m KOH aqueous electrolyte solution. Figure S19 in the Supporting Information presents the electrochemical performance of pristine CNTF and its contribution is completely negligible for our as‐fabricated negative electrodes. Additionally, the pristine CNTF used as negative electrode indicates a typical double electrode layer capacitive material. Comparison of the CV curves of the TiN NWAs@VN NSs/CNTF, VN NSs/CNTF, and TiN NWAs/CNTF electrodes acquired at a scan rate of 100 mV s^−1^ in the potential window from −1.2 to −0.2 V revealed the substantially higher capacitance of the TiN NWAs@VN NSs compared with the TiN NWAs and VN NSs (Figure 5a). Furthermore, the results of the GCD measurements at the same current density (1 mA cm^−2^) for the three electrodes demonstrated that the discharge time for the TiN NWAs@VN NSs/CNTF electrode was significantly longer than that for the other two electrodes (Figure 5b). The improved electrochemical performance of the TiN NWAs@VN NSs was ascribed to the high electrical conductivity of the TiN core and synergistic effects of the well‐aligned TiN NWAs and VN NSs. The CV curves of the TiN NWAs possessed similar rectangular shapes, demonstrating their ideal capacitance behavior and rapid charge/discharge characteristics (Figure S20a, Supporting Information). Upon increasing the scan rate from 50 to 500 mV s^−1^, the CV profiles still maintained a rectangular shape without severe polarization, which demonstrates the outstanding reversibility and excellent rate performance of the TiN NWAs. The almost symmetric GCD curves for the TiN NWAs/CNTF electrode also indicated fast charge/discharge characteristics and electrochemical reversibility (Figure S20b, Supporting Information). These results further demonstrate the superiority of the TiN NWAs as the core electrode material. Furthermore, the CV curves of the VN NSs/CNTF electrode possessed approximately rectangular shapes, with two pairs of concave/convex peaks at potentials of −0.8 to −0.6 V and −0.6 to −0.4 V (Figure S21a, Supporting Information), indicating a pseudocapacitive charge‐storage mechanism. Even at a scan rate of 200 mV s^−1^, the curve still retained a rectangular shape, further demonstrating the promising rate capability of the VN NSs, which is in accordance with the GCD test results (Figure S21b, Supporting Information). This result demonstrates the enormous advantage of using the VN NSs as the shell layer. The electrochemical characteristics of the three electrodes were further investigated by EIS measurements and the corresponding Nyquist plots are presented in Figure 5c. We also measured the compacted electrical resistance of the three metallic compound powders for comparison, as shown in Figure S22 in the Supporting Information. According to the magnified Nyquist plots (inset in Figure 5c), the TiN NWAs/CNTF exhibited the lowest equivalent series resistance (*R*_s_) of 8.53 Ω, whereas the VN NSs/CNTF exhibited the highest *R*_s_ of 15.76 Ω. It is worth noting that the use of the highly conductive TiN NWAs as the secondary support significantly decreased the *R*_s_ (11.62 Ω) of the electrode with the core–shell heterostructure, further demonstrating the superiority of the TiN NWAs as a core material. As shown in Figure 5d, upon increasing the scan rate from 10 to 100 mV s^−1^, the CV curves still retained an approximately rectangular shape without severe polarization, indicating outstanding reversibility and excellent rate performance for the TiN NWAs@VN NSs. Additionally, the symmetric shapes of the GCD curves at various current densities also indicate excellent reversibility (Figure 5e). The specific capacitances of the three electrodes at various current densities (1–10 mA cm^−2^) are summarized in Figure 5f. The TiN NWAs@VN NSs/CNTF electrode exhibited the highest specific capacitance of 1255.2 mF cm^−2^ at a current density of 1 mA cm^−2^, whereas the other two electrodes showed a lower specific capacitance. Furthermore, even at the high current density of 10 mA cm^−2^, the TiN NWAs@VN NSs/CNTF electrode retained a high specific capacitance (854.6 mF cm^−2^) corresponding to 68.1% of the specific capacitance at a current density of 1 mA cm^−2^, demonstrating its high rate performance compared with the one‐component electrode materials. These values considerably exceed previously reported works, as presented in Table S2 in the Supporting Information. These results indicate that the use of TiN NWAs as the core material can significantly improve the stability of the VN NSs as the shell layer. A comparison of the specific capacitance calculated by volume and rate performance of the three electrodes is presented in Figure S23 in the Supporting Information. The improved stability was ascribed to the TiN NWAs, which offer fast electronic channels and stabilize the structure of the active material during prolonged electrochemical reactions. Overall, the as‐fabricated CoNiO_2_ NWAs@Ni(OH)_2_ NSs/CNTF and TiN NWAs@VN NSs/CNTF electrodes are undoubtedly promising cathode and anode materials, respectively, for the fabrication of high‐energy‐density FASCs.

**Figure 6:**
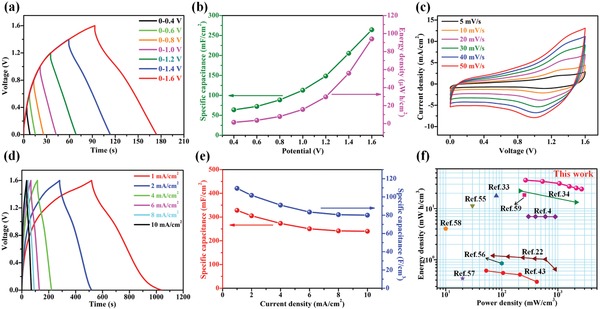
a) GCD curves for the FASC device acquired at various voltages (0.4–1.6 V) with a current density of 5 mA cm^−2^. b) Specific capacitance and energy density calculated from the GCD curves obtained at 5 mA cm^−2^ as a function of the voltage window. c) CV curves for the device obtained at various scan rates between 5 and 50 mV s^−1^. d) GCD curves for the device acquired at various current densities between 1 and 10 mA cm^−2^. e) Specific capacitances of the device calculated from the GCD curves as a function of the current density. f) Volumetric energy and power densities of the device compared with those of previously reported devices.

An FASC device was next assembled to evaluate the performance of CoNiO_2_ NWAs@Ni(OH)_2_ NSs/CNTF and TiN NWAs@VN NSs/CNTF as cathode and anode materials for practical applications (Figure S24, Supporting Information). To obtain good charge‐storage properties, it is necessary to balance the electric charges stored on the cathode and anode. Thus, based on the specific capacitance and voltage windows of the two materials, the areas of the cathode and anode were optimized using the following equation

(2) $$\frac{A^{+}}{A^{-}}=\frac{C^{-}\times \Delta V^{-}}{C^{+}\times \Delta V^{+}}$$

where *A* ^+^, *C* ^+^, and Δ*V* ^+^ are the area, specific capacitance, and operating voltage of the CoNiO_2_ NWAs@Ni(OH)_2_ NSs/CNTF cathode, respectively, and *A* ^−^, *C* ^−^, and Δ*V* ^−^ are area, specific capacitance, and operating voltage of the TiN NWAs@VN NSs/CNTF anode, respectively. Figure S25 in the Supporting Information shows that the device exhibited an optimal working potential window of 1.6 V. Furthermore, the GCD curves of the device at a current density of 5 mA cm^−2^ were symmetrical with a triangular shape, even at the high working voltage of 1.6 V (**Figure** **6**a), indicating that the device possessed ideal capacitive behavior and low equivalent series resistance. Figure 6b shows the specific capacitances and energy densities of the FASC device, which were calculated from the GCD curves at 5 mA cm^−2^. The calculated specific capacitance and areal energy density dramatically increased from 63.8 to 264.2 mF cm^−2^ and from 0.8 to 85.8 µWh cm^−2^, respectively, when the working potential was increased from 0.4 to 1.6 V. Figure 6c shows the CV curves of the as‐assembled FASC device at various scan rates ranging from 5 to 50 mV s^−1^. The CV curves exhibit electric double‐layer capacitive and battery‐type behavior, and the CV curve shape is retained even at the high scan rate of 50 mV s^−1^, indicating the remarkable reversibility of the device. The nearly symmetrical shapes of all of the GCD curves indicate almost equal charge/discharge times, demonstrating high Coulombic efficiency and Faradaic reversibility (Figure 6d). The specific capacitance values of the device calculated from the GCD curves as a function of the current density are plotted in Figure 6e. The as‐assembled FASC device exhibited a remarkable specific capacitance of 109.4 F cm^−3^ (328.3 mF cm^−2^) at the typical discharge current density of 1 mA cm^−2^ and retained a specific capacitance of 80.0 F cm^−3^ (240.0 mF cm^−2^) even at the high current density of 10 mA cm^−2^, demonstrating an excellent rate capability (73.13%). The energy density and power density are the two crucial parameters for evaluating the practical applications of SCs. The relationship between the energy density and the power density of the FASC device is presented in Figure 6f alongside the corresponding values for previously reported devices. The as‐assembled FASC device was able to store a maximum volumetric energy density of 36.0 mWh cm^−3^ with a volumetric power density of 266.7 mW cm^−3^ at a current density of 1 mA cm^−2^. The device still displayed a volumetric energy density of 24.1 mWh cm^−3^ at the highest tested power density of 2666.7 mW cm^−3^, demonstrating an outstanding energy‐storage performance. The areal energy and power densities of the FASC device are presented in Figure S26 in the Supporting Information. The achieved energy densities of our FASC device rival or indeed exceed those of previously reported devices based on a single electrode with a solitary or core–shell structure.^4^, ^22^, ^33^, ^34^, ^43^, ^55^, ^56^, ^57^, ^58^, ^59^ Furthermore, the as‐assembled FASC device possessed excellent cycling stability (Figure S27, Supporting Information). These characteristics of our device were ascribed to the synergistic effects obtained by combining the TiN NWAs@VN NSs and CoNiO_2_ NWAs@Ni(OH)_2_ NSs core–shell heterostructures.

**Figure 7:**
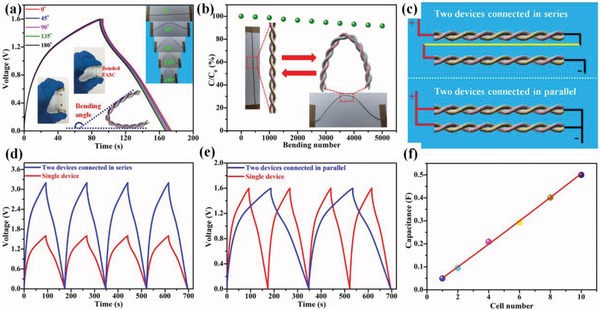
a) GCD curves for the as‐assembled FASC device measured at a current density of 5 mA cm^−2^ under different bending angles. b) Normalized capacitance of the as‐obtained FASC device over 5000 bending cycles at a bending angle of 90°. c) Schematic illustrations of two FASC devices connected in series and in parallel. d,e) GCD curves for two FASC devices connected in series and in parallel at 5 mA cm^−2^. f) Relationship between total device capacitance and number of FASC devices connected in parallel.

The ability to withstand severe bending is a prerequisite for the practical use of flexible SC devices in portable and wearable electronics.^8^, ^14^, ^21^, ^23^ As shown in **Figure** **7**a, the observed variations in the GCD curves were negligible under various bending angles from 0° to 180° at a current density of 5 mA cm^−2^, demonstrating the excellent flexibility of the as‐fabricated FASC device. In addition, the device still retained 91.5% of its specific capacitance after 5000 bending cycles, as shown by the EIS results presented in Figure S28 in the Supporting Information, further demonstrating the robust mechanical performance of the FASC device. To satisfy the potential or power requirements of practical applications, FASC devices must be connected either in series or in parallel as depicted in Figure 7c. The charge/discharge operating voltage window and charge/discharge times were doubled when two of the FASC devices were connected in series and in parallel, respectively, as shown in Figure 7d,e, demonstrating the high stability of the integrated FASC device. Additionally, the total capacitance increased linearly with the number of devices when multiple FASC devices (from 1 to 10) were connected in parallel, demonstrating excellent scalability (Figure 7f). To further demonstrate the potential applications of the integrated FASC devices, two FASC devices connected in series were used to power a red light‐emitting diode (1.8–2.2 V) (Figure S29, Supporting Information), and the integrated device also demonstrated excellent flexibility (Figure S30, Supporting Information).

## 3 Conclusion

A facile and cost‐effective synthetic strategy was used to fabricate novel 3D well‐aligned hierarchical CoNiO_2_ NWAs@Ni(OH)_2_ NSs and TiN NWAs@VN NSs core–shell heterostructures on CNTFs for use as cathode and anode materials, respectively. Owing to the enhanced electrical conductivity of the core materials (CoNiO_2_ and TiN NWAs) and the synergistic effects of the core and shell materials, the as‐fabricated CoNiO_2_@Ni(OH)_2_ NWAs/CNTF exhibited ultrahigh capacity of 0.674 mAh cm^−2^ and TiN@VN NWAs/CNTF electrodes showed high specific capacitances of 1255.2 mF cm^−2^. A high‐performance FASC device based on the two electrodes coated with KOH–poly(vinyl alcohol) (PVA) gel electrolyte was then assembled. This FASC device exhibited a maximum work voltage of 1.6 V, an extraordinary specific capacitance of 109.4 F cm^−3^, and a prominent energy density of 36.0 mWh cm^−3^, which are far superior to previously reported values for all‐solid‐state FSC devices. Moreover, the FASC device exhibited excellent flexibility, with the capacitance remaining essentially unchanged after bending from 0° to 180° and only decreasing to 91.5% even after 5000 cycles of bending to 90°. The work voltage and capacitance doubled upon connecting two of the FASC devices in series and in parallel, respectively. Overall, the successful development of the ultrahigh‐capacity cathode material CoNiO_2_ NWAs@Ni(OH)_2_ NSs and the anode material TiN NWAs@VN NSs with core–shell heterostructures provides a convenient and effective strategy for the design of new electrode materials for next‐generation wearable supercapacitors.

## 4 Experimental Section

*Preparation of CoNiO_2_ NWAs@Ni(OH)_2_ NSs/CNTF*: The CoNiO_2_ NWAs on CNTFs were synthesized via a simple hydrothermal method and subsequent high‐temperature calcination. Prior to the growth of the samples on the CNTFs, the CNTFs were pretreated with O_2_ plasma at a power of 150 W for 8 min. In a typical experiment, 0.875 mmol of Ni(NO_3_)_2_·6H_2_O and Co(NO_3_)_2_·6H_2_O, 2 mmol of NH_4_F, and 8 mmol of CO(NH_2_)_2_ were dissolved in 30 mL of deionized (DI) water and stirred vigorously to obtain a uniform solution. The CNTFs were immersed in the solution and the mixture was transferred to a 50 mL Teflon‐lined stainless steel autoclave. The autoclave was then sealed and maintained at 120 °C for 3 h. After allowing the sample to cool to room temperature, the CNTFs with the grown sample were washed several times with DI water, dried for 12 h at 60 °C, and finally annealed at 300 °C for 2 h in air at a heating rate of 5 °C min^−1^ to afford the CoNiO_2_ NWAs/CNTFs, and the mass loading of the CoNiO_2_ NWAs is measured to be 0.81 mg cm^−2^. The synthesized CoNiO_2_ NWAs/CNTFs were suspended in a homogeneous solution containing 10 g of NiSO_4_·6H_2_O, 2 g of K_2_S_2_O_8_, 5 mL of aqueous ammonia (28%), and 100 mL of DI water for 30 min. Finally, the positive electrode CoNiO_2_ NWAs@Ni(OH)_2_ NSs/CNTF was washed several times with DI water and dried at 60 °C for 10 h under vacuum (mass loading: 1.23 mg cm^−2^). Ni(OH)_2_ NSs/CNTFs were also prepared by the same method for comparison.

*Preparation of TiN NWAs@VN NSs/CNTF*: The TiN NWAs on CNTFs were synthesized by a facile hydrothermal method followed by high‐temperature nitrogenization. First, the CNTFs were immersed in 0.2 mol L^−1^ of titanium tetrachloride (TiCl_4_) aqueous solution containing 2.2 mL of TiCl_4_ and 100 mL of DI water and then maintained at 100 °C for 1 h. The resulting CNTFs were dried at 60 °C for 2 h and then annealed in air at 350 °C for 1 h to form a seeded layer on the surface. Second, the CNTFs with the seeded layer were immersed in a homogeneous solution containing 0.75 mL of TiCl_4_, 45 mL of 36–38 wt% hydrochloric acid solution, and 45 mL of DI water. This mixture was transferred to a 100 mL Teflon‐lined stainless steel autoclave and maintained at 150 °C for 6 h to coat the TiO*_x_* NWAs on the CNTFs. The resulting CNTFs were repeatedly washed with DI water and dried under vacuum at 60 °C for 12 h. Subsequently, the as‐dried CNTFs were converted to the TiN NWAs/CNTFs by annealing at 800 °C for 8 h under an atmosphere of ammonia gas at a flux of 200 sccm with argon as the shielding gas at a flux of 200 sccm (mass loading: 0.5 mg cm^−2^). The TiN NWAs/CNTFs were then immersed in a solution of 0.3 mL of vanadium oxytriisopropoxide in 45 mL of isopropanol and the resulting mixture was stirred for 30 min prior to transfer into a 50 mL Teflon‐lined stainless steel autoclave. The autoclave was then sealed and maintained at 200 °C for 10 h. After allowing the sample to cool to room temperature, the obtained CNTFs were washed with ethanol and dried under vacuum at 60 °C for 12 h. Finally, the TiN NWAs/CNTFs coated with VO*_x_* were annealed at 600 °C for 6 h under an atmosphere of ammonia gas (200 sccm) and argon (200 sccm) to obtain the VN NSs (mass loading: 0.92 mg cm^−2^). VN NSs/CNTFs were also prepared by the same method for comparison.

*Assembly of CoNiO_2_ NWAs@Ni(OH)_2_ NSs/CNTF // TiN NWAs@VN NSs/CNTF FASCs*: The FASCs were assembled using the hierarchical core–shell structured CoNiO_2_ NWAs@Ni(OH)_2_ NSs/CNTF as the positive electrode and TiN NWAs@VN NSs/CNTF as the negative electrode, and KOH–PVA as the gel electrolyte. The gel electrolyte was prepared by blending 11.2 g of KOH and 10 g of PVA in 100 mL of DI water under intense stirring at 95 °C for 2 h until the solution became clear. The CoNiO_2_ NWAs@Ni(OH)_2_ NSs/CNTF and TiN NWAs@VN NSs/CNTF electrodes were immersed in the KOH–PVA gel electrolyte for 10 min and subsequently dried at 60 °C for 2 h, which was repeated twice. Finally, the all‐solid‐state FASC device was successfully assembled by twisting the two electrodes together followed by drying at 60 °C for 12 h to ensure complete curing of the KOH–PVA gel electrolyte.

## Conflict of Interest

The authors declare no conflict of interest.

## Supporting information

Supplementary
