# Supplementary material for: All Hierarchical Core–Shell Heterostructures as Novel Binder‐Free Electrode Materials for Ultrahigh‐Energy‐Density Wearable Asymmetric Supercapacitors
Source: Adv Sci (Weinh). 2018 Nov 12;6(2):1801379. doi: 10.1002/advs.201801379 (PMC6343089; doi:10.1002/advs.201801379)
Supplement: Supplementary file 1 — Supplementary [file ADVS-6-1801379-s001.pdf]

Copyright WILEY-VCH Verlag GmbH & Co. KGaA, 69469 Weinheim, Germany, 2018.

ADVANCED SCIENCE
Open Access

Supporting Information

for *Adv. Sci.,* DOI: 10.1002/advs.201801379

All Hierarchical Core–Shell Heterostructures as Novel Binder-Free Electrode Materials for Ultrahigh-Energy-Density Wearable Asymmetric Supercapacitors

*Qiulong Li, Qichong Zhang, Juan Sun, Chenglong Liu, Jiabin Guo, Bing He, Zhenyu Zhou, Ping Man, Chaowei Li, Liyan Xie, and Yagang Yao**

Supporting Information

All Hierarchical Core–Shell Heterostructures as Novel Binder-Free Electrode Materials for Ultrahigh-Energy-Density Wearable Asymmetric Supercapacitors

Qiulong Li[+], Qichong Zhang[+], Juan Sun, Chenglong Liu, Jiabin Guo, Bing He, Zhenyu Zhou, Ping Man, Chaowei Li[a], Liyan Xie, Yagang Yao*

Materials

Nickel nitrate hexahydrate ($(Ni(NO_3)_2 \cdot 6H_2O$, 98%), ammonium fluoride (NH_4F, 96%), urea ($CO(NH_2)_2$, 99%), nickel sulfate hexahydrate ($NiSO_4 \cdot 6H_2O$, 98.5%), potassium persulfate ($K_2S_2O_8$, 99.5%), ammonia water ($NH_3 \cdot H_2O$, 28%), hydrochloric acid (HCl, 36-38%), isopropanol alcohol (C_3H_8O, 99.7%), and potassium hydroxide (KOH, 85%) were obtained from Sinopharm Chemical Reagent, China. Cobalt nitrate hexahydrate ($Co(NO_3)_2 \cdot 6H_2O$, 98.5%), titanium tetrachloride ($TiCl_4$, 99%), and PVA ($[CH_2CH\ (OH)]_n$, n = 1799) were purchased from Aladdin. Vanadium oxytriisopropoxide ($C_9H_{21}O_4V$, 97%) was purchased from Tstachi (Shanghai) into Industrial Development Co., Ltd, (Shanghai, China). Each of these chemicals was used directly without any further purification. Carbon nanotube fibers (CNTFs) were fabricated with a floating catalyst chemical vapor deposition method followed by shrinking with ethanol.

Prepartion of carbon nanotube fibers (CNTFs)

The CNTFs were fabricated by two processes including of floating catalyst chemical vapor deposition (FCCVD) method and twisting method. The pristine CNT strip used for fabrication of CNTFs was synthesized at 1300 °C using ethanol and ferrocene as carbon source and catalyst via FCCVD process. The typical thickness, mechanical strength, and electrical conductivity of the pristine CNT strip are around 10 μm, 30-50 MPa, and 10^5 S/m, respectively. The CNTFs were fabricated by twisting a CNT strip via a fast and scale process and were used for current collector.

Materials characterization

The morphologies of the as-prepared samples were characterized by scanning electron microscopy (SEM; Hitachi S-4800, 5 kV). The microstructures of the samples were observed by transmission electron microscopy (TEM; FEI Tecnai G2 F20 S-Twin), and high-resolution TEM images were acquired on an FEI Tecnai G2 20 high-resolution transmission electron microscope operating at an acceleration voltage of 200 kV. The chemical compositions of the samples were analyzed on an ESCALAB MKII X-ray photoelectron spectrometer (XPS) using non-monochromatized Mg Kα X-rays as the excitation source. X-ray diffraction (XRD) patterns were acquired on a Rigaku D/MAX2500 V system using Cu Kα radiation (λ = 1.5418 Å).

Electrochemical Performance Measurements

The electrochemical performance of the bare CNTF and fabricated samples was evaluated by galvanostatic charge/discharge (GCD), cyclic voltammetry (CV), and electrochemical impedance spectroscopy (EIS) measurements on an electrochemical workstation (CHI 760E, Chenhua) using a three-electrode configuration in 3 M KOH aqueous solution. The fabricated electrode materials, Pt wire and Ag/AgCl were used as the working, counter and reference electrodes, respectively. The EIS measurements were assumed between 10^{-2}-10^{5} Hz with a voltage amplitude of 5 mV and open-circuit potential. The specific capacitance (C), energy density (E), and power density (P) were calculated according to the following equations:

$$C_A = \frac{I\Delta t}{A\Delta V} \quad (1)$$

$$E_A = \frac{I\int_{t1}^{t2} U(t)dt}{A} \quad (2)$$

$$P_A = \frac{E_A}{\Delta t} \quad (3)$$

$$C_V = \frac{I\Delta t}{V\Delta V} \quad (4)$$

$$E_V = \frac{I\int_{t1}^{t2} U(t)dt}{V} \quad (5)$$

$$P_V = \frac{E_V}{\Delta t} \quad (6)$$

Where A and V are the total area and volume of the as-assembled fiber-shaped asymmetric supercapacitors (FASCs), respectively. I and U(t) are the discharge current and real-time voltage, respectively. t_1 and t_2 are the initial and end time of the GCD discharge curves, respectively. The cycle life tests were conducted with GCD measurements with a constant current density of 5 mA/cm^2 for 8,000 cycles.

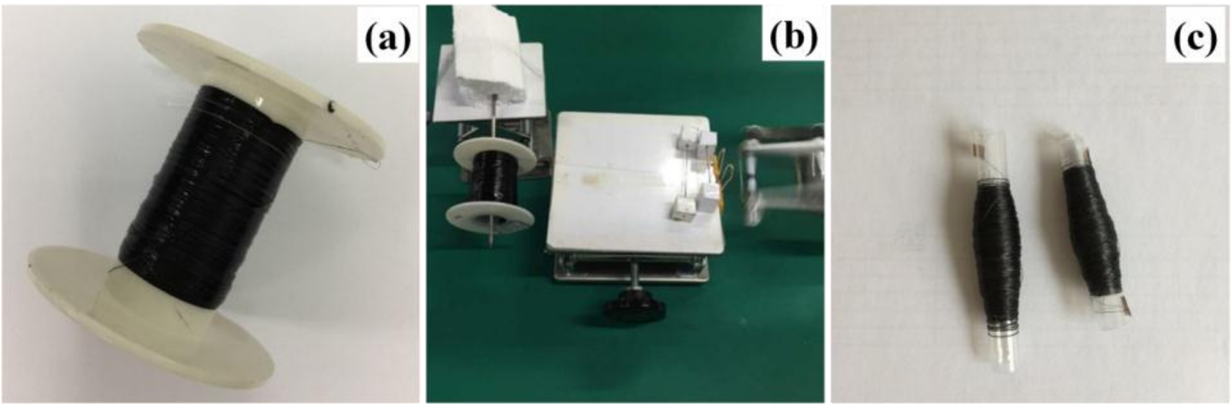

Figure S1 (a) Photograph of 60-m CNT strip. (b) Photograph of the experimental setup to prepare CNTF from a CNT strip. (c) Photograph of 60-m CNTF wrapped around a plastic tube.

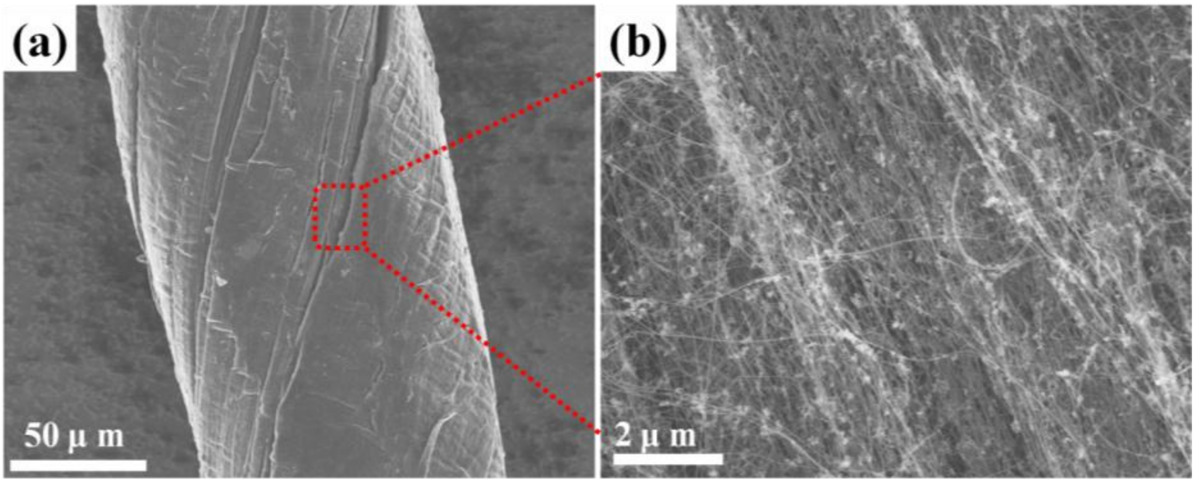

Figure S2 (a, b) SEM images of bare CNTF at different magnifications.

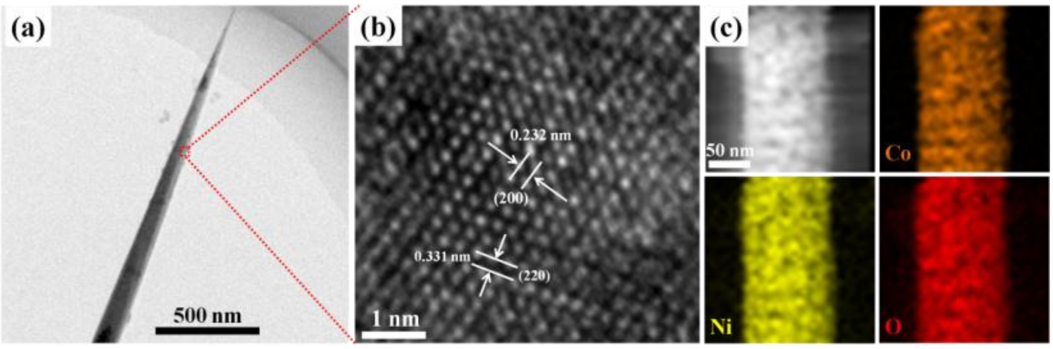

Figure S3 (a) Low-magnification TEM image of single $CoNiO_2$ nanowire. (b) High-magnification of the red rectangle in panel a. (c) TEM image of the $CoNiO_2$ nanowire and the corresponding EDS element mapping images.

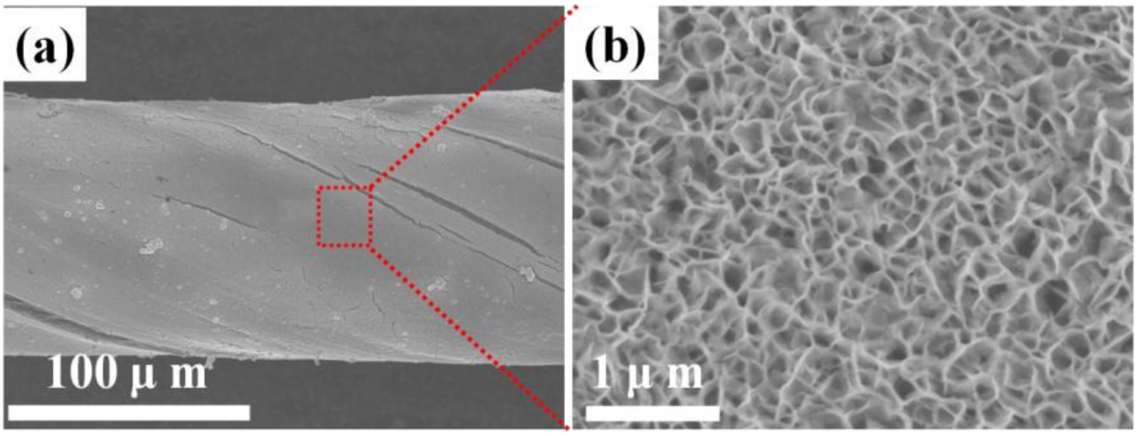

Figure S4 (a, b) SEM images of the $Ni(OH)_2$ NSs/CNTF at different magnifications.

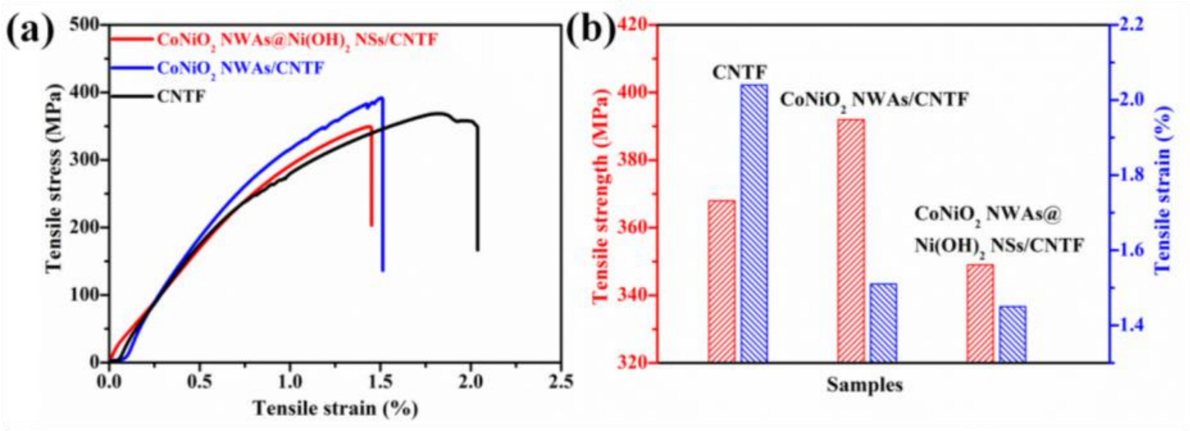

Figure S5 (a) Comparison of stress-strain curves of pristine CNTF, $CoNiO_2$ NWAs/CNTF, and $CoNiO_2$ NWAs@$Ni(OH)_2$ NSs/CNTF. (b) Comparison of tensile strength and tensile strain of pristine CNTF, $CoNiO_2$ NWAs/CNTF and $CoNiO_2$ NWAs@$Ni(OH)_2$ NSs/CNTF.

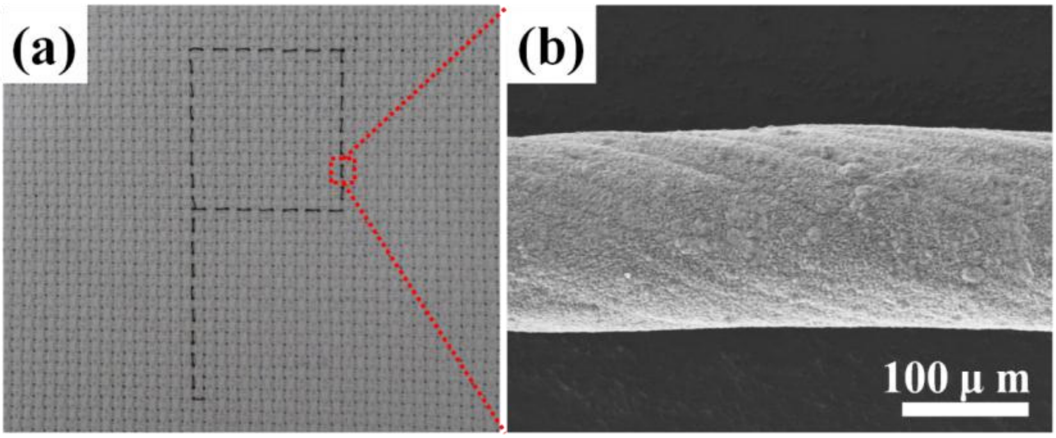

Figure S6 (a) Optical image of the textiles for the positive electrode $CoNiO_2$ NWAs@$Ni(OH)_2$ NSs/CNTF as a "P" shape. (b) SEM image of the $CoNiO_2$ NWAs@$Ni(OH)_2$ NSs/CNTF.

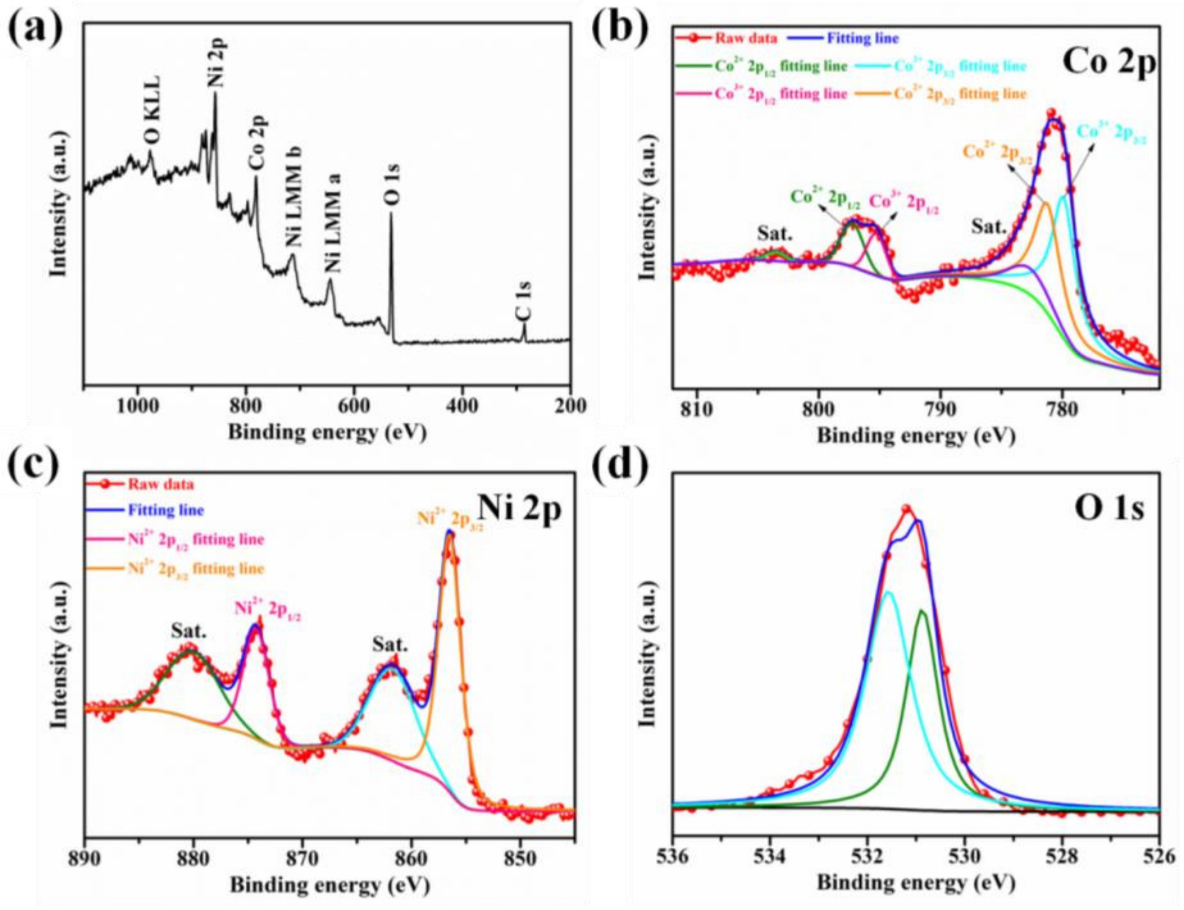

Figure S7 (a) XPS survey spectrum and (b-d) high-resolution spectra of Co (b), Ni (c), and O (d), presented in the $CoNiO_2$ NWAs@$Ni(OH)_2$ NSs/CNTF.

The survey spectrum in Figure S7(a) indicates that the $CoNiO_2$ NWAs@$Ni(OH)_2$ NSs/CNTF sample consists of Co, Ni, O, and C, in which the element C comes from the substrate CNTF and amorphous carbon derived from the annealing process. Therefore, Co, Ni, and O are the main chemical compositions in the near surface of the $CoNiO_2$ NWAs@$Ni(OH)_2$ NSs/CNTF samples. Figure S7(b)-(d) displays the typical Co 2p, Ni 2p, and O 1s narrow scan of the $CoNiO_2$ NWAs@$Ni(OH)_2$ NSs/CNTF. The Co $2p_{1/2}$ region shows two peaks at 797.3 and 795.3 eV, and the Co $2p_{3/2}$ region shows two peaks at 781.5 and 778.3 eV (Figure S7(b)). Meanwhile, two satellite (denoted as Sat.) lines associated with Co 2p are also present. Two typical

peaks centered at 873.7 and 855.6 eV are observed in Figure S7(c) and are in accordance with Ni $2p_{1/2}$ and Ni $2p_{3/2}$ of $Ni(OH)_2$, respectively. Moreover, two Sat. lines in connection with Ni 2p also appear. In the O 1s region (Figure S7(d)), binding energy at 531.6 eV are identified as OH groups and oxygen containing group of C-O derived from the CNTF. Furthermore, the O1s peak located at 530.3 eV corresponds to the oxygen species forming oxide with nickel and cobalt elements.

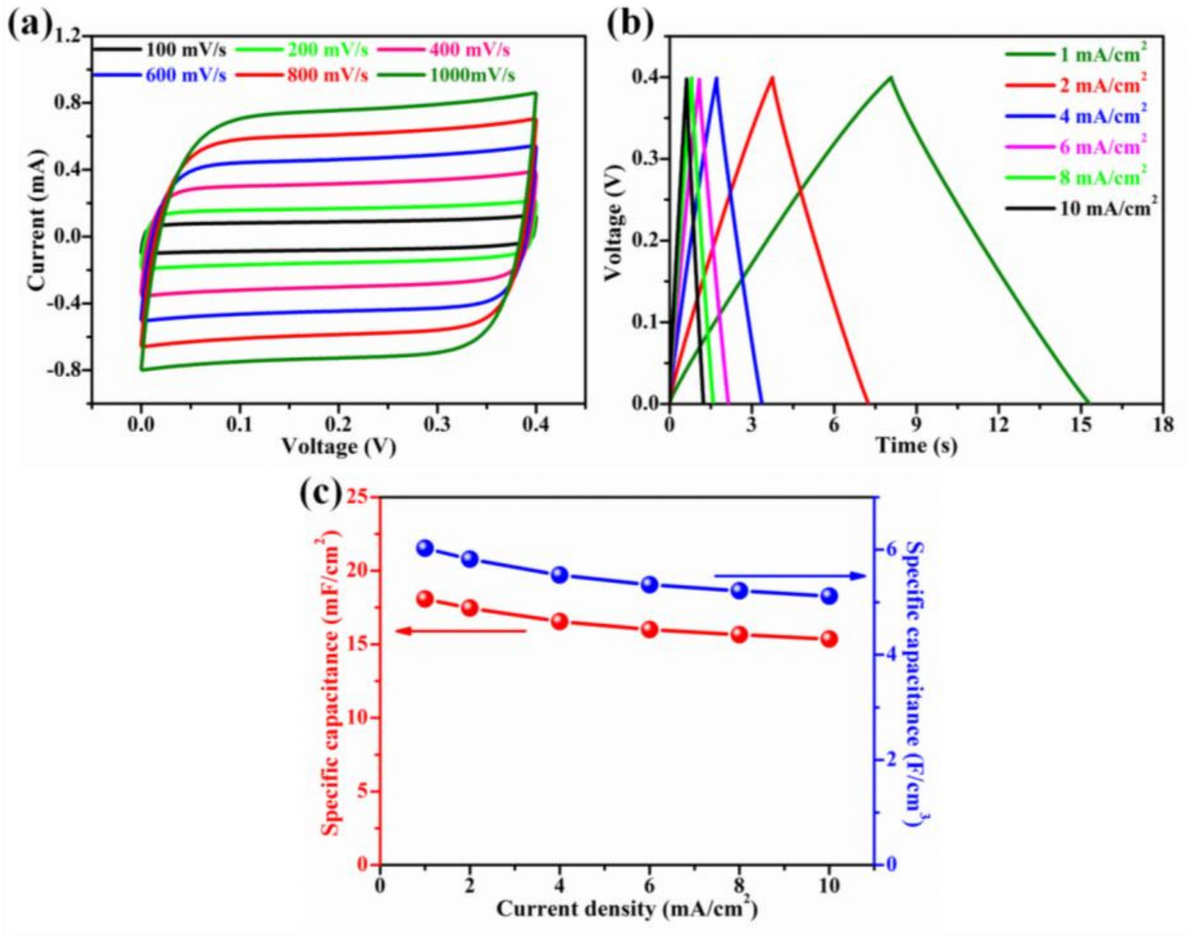

Figure S8 (a) CV curves of the bare CNTF electrode (used as positive electrode) in the potential window ranging from 0 to 0.4 V at various scan rates. (b) GCD curves of bare CNTF electrode in the potential window ranging from 0 to 0.4 V at different current densities. (c) Specific capacitances of the bare CNTF calculated from the corresponding discharge curves as a function of current density.

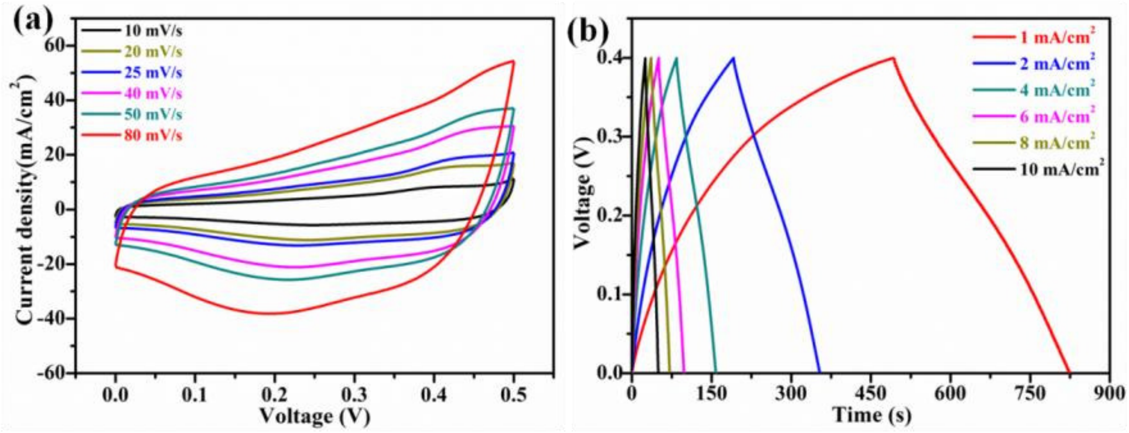

Figure S9 (a) CV curves of the $CoNiO_2$ NWAs/CNTF electrode at different scan rates. (b) GCD curves of the $CoNiO_2$ NWAs/CNTF electrode at different current densities.

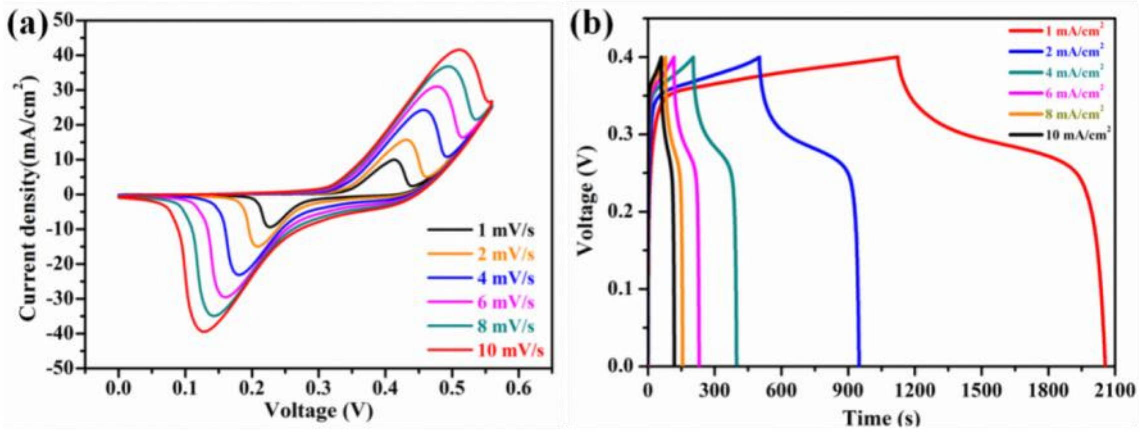

Figure S10 (a) CV curves of the $Ni(OH)_2$ NSs/CNTF electrode at different scan rates.

(b) GCD curves of the $Ni(OH)_2$ NSs/CNTF electrode at different current densities.

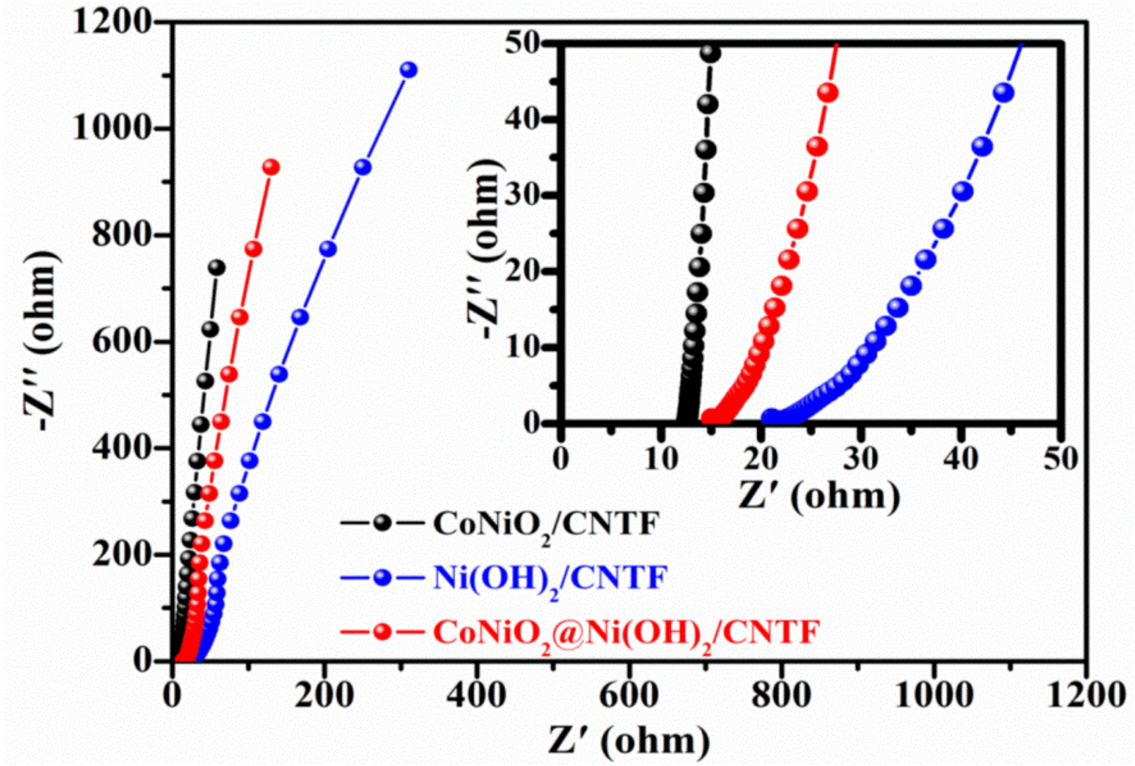

Figure S11 Nyquist plot of the $CoNiO_2$ NWAs@$Ni(OH)_2$ NSs/CNTF, $CoNiO_2$ NWAs/CNTF and $Ni(OH)_2$ NSs/CNTF electrodes at frequencies ranging from 10^{-2} to 10^5 Hz with a voltage amplitude of 5 mV at open-circuit potential (the inset presents the partially enlarged Nyquist plot).

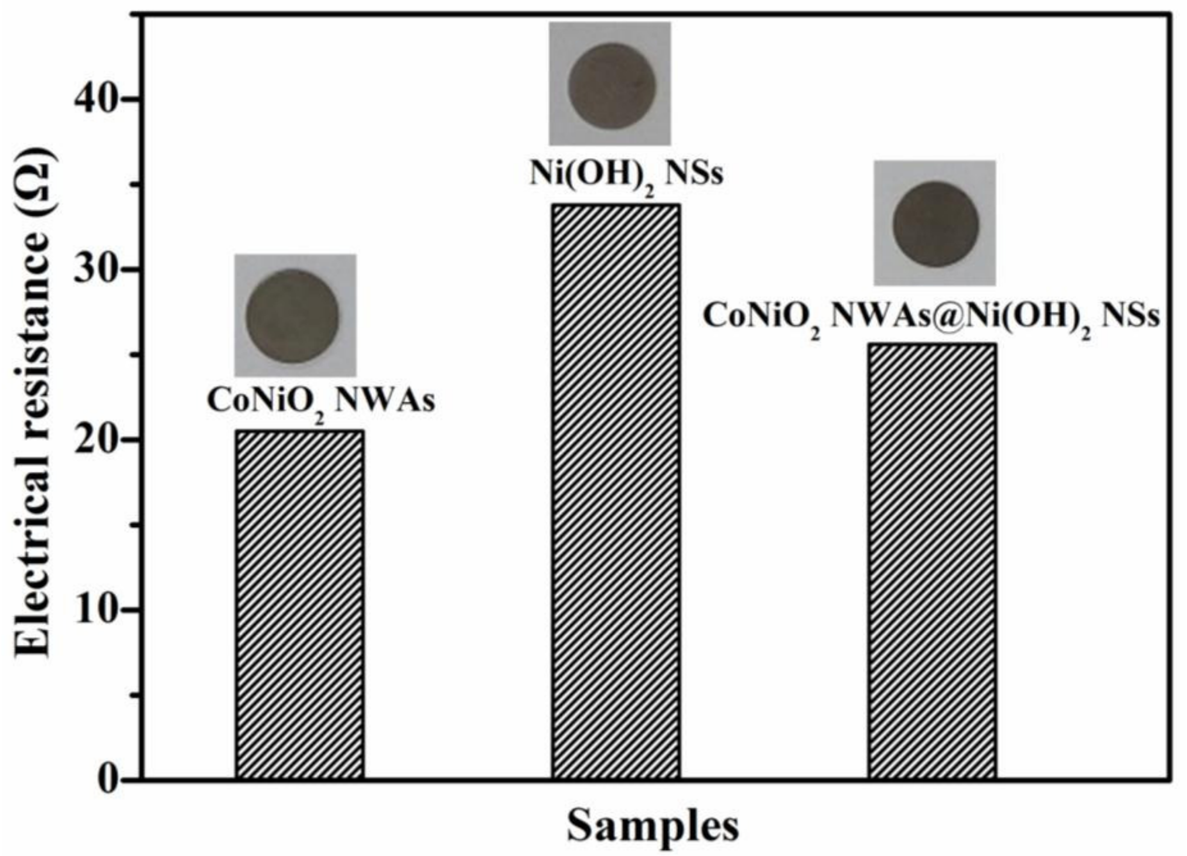

Figure S12 The compacted electrical resistance of the as-synthesized $CoNiO_2$ NWAs, $Ni(OH)_2$ NSs, $CoNiO_2$ NWAs@$Ni(OH)_2$ NSs powders.

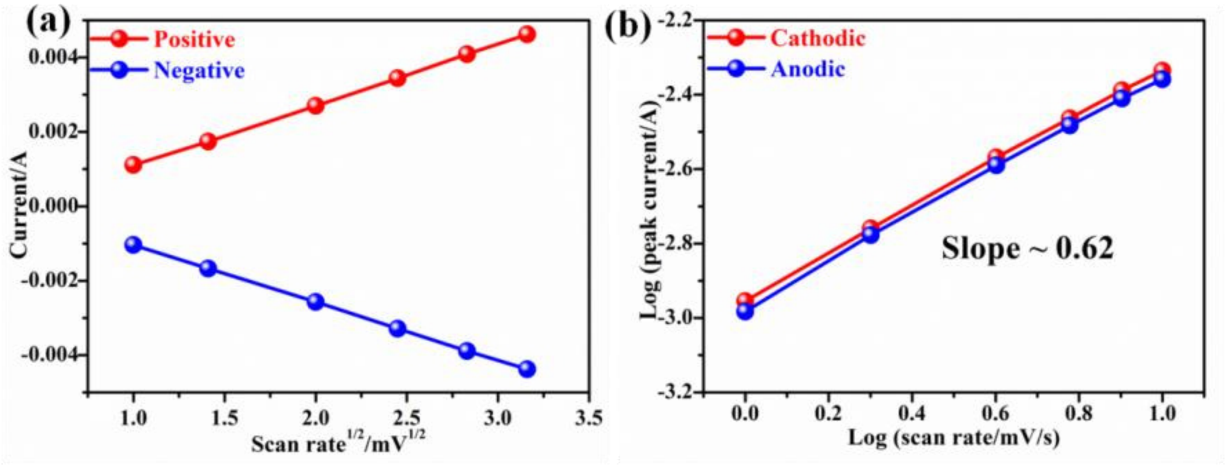

Figure S13 (a) Plots of the cathodic and anodic peaks currents versus the square root of the scan rates and (b) Linear fitting of the peak current versus scan rate for the cathodic and anodic peaks in the CV curves (Figure S6a) of the $Ni(OH)_2$ NSs/CNTF electrode.

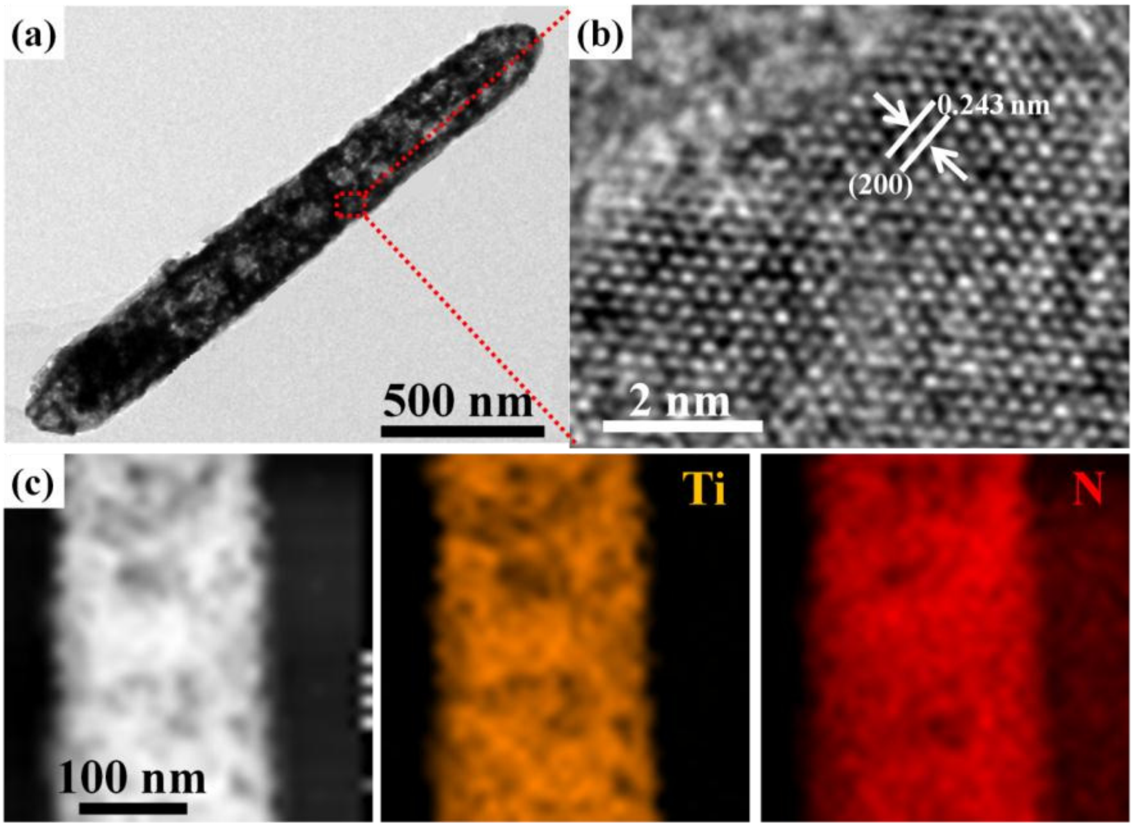

Figure S14 (a) Low-magnification TEM image of single TiN nanowire. (b) High-magnification of the red rectangle in panel a. (c)TEM image of the TiN nanowire and the corresponding EDS element mapping images.

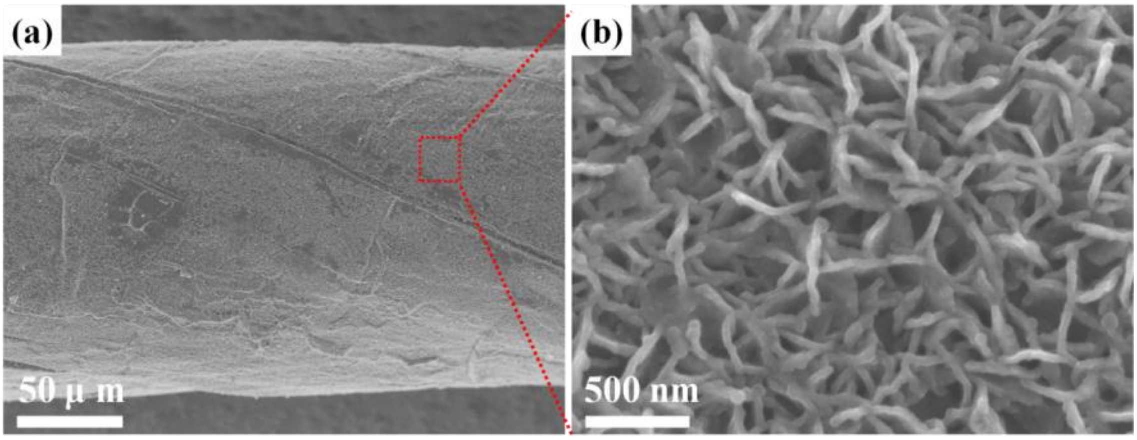

Figure S15 (a) SEM image of the VN NSs grown on the CNTF surface at low magnification. (b) SEM image of the VN NSs/CNTF at high magnification.

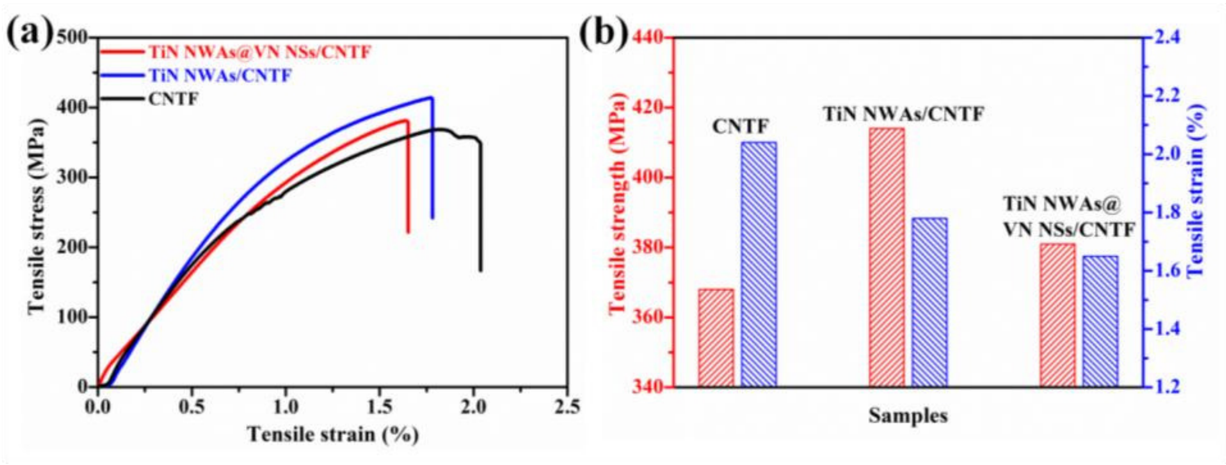

Figure S16 (a) Comparison of stress-strain curves of pristine CNTF, TiN NWAs/CNTF, and TiN NWAs@VN NSs/CNTF. (b) Comparison of tensile strength and tensile strain of pristine CNTF, TiN NWAs/CNTF, and TiN NWAs@VN NSs/CNTF.

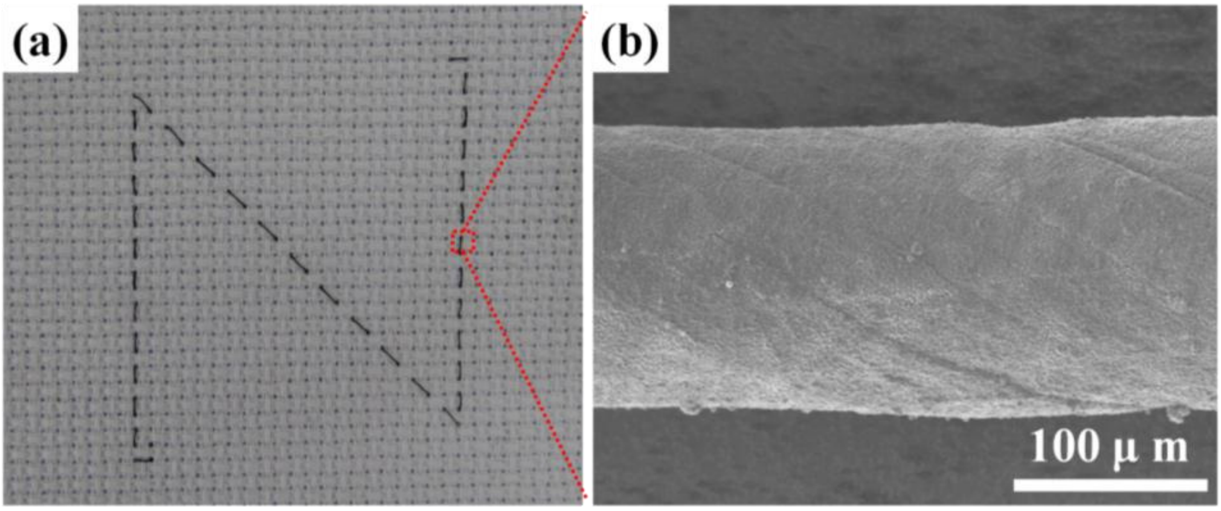

Figure S17 (a) Optical image of the textiles for the negative electrode TiN NWAs@VN NSs/CNTF as an "N" shape. (b) SEM image of the TiN NWAs@VN NSs/CNTF.

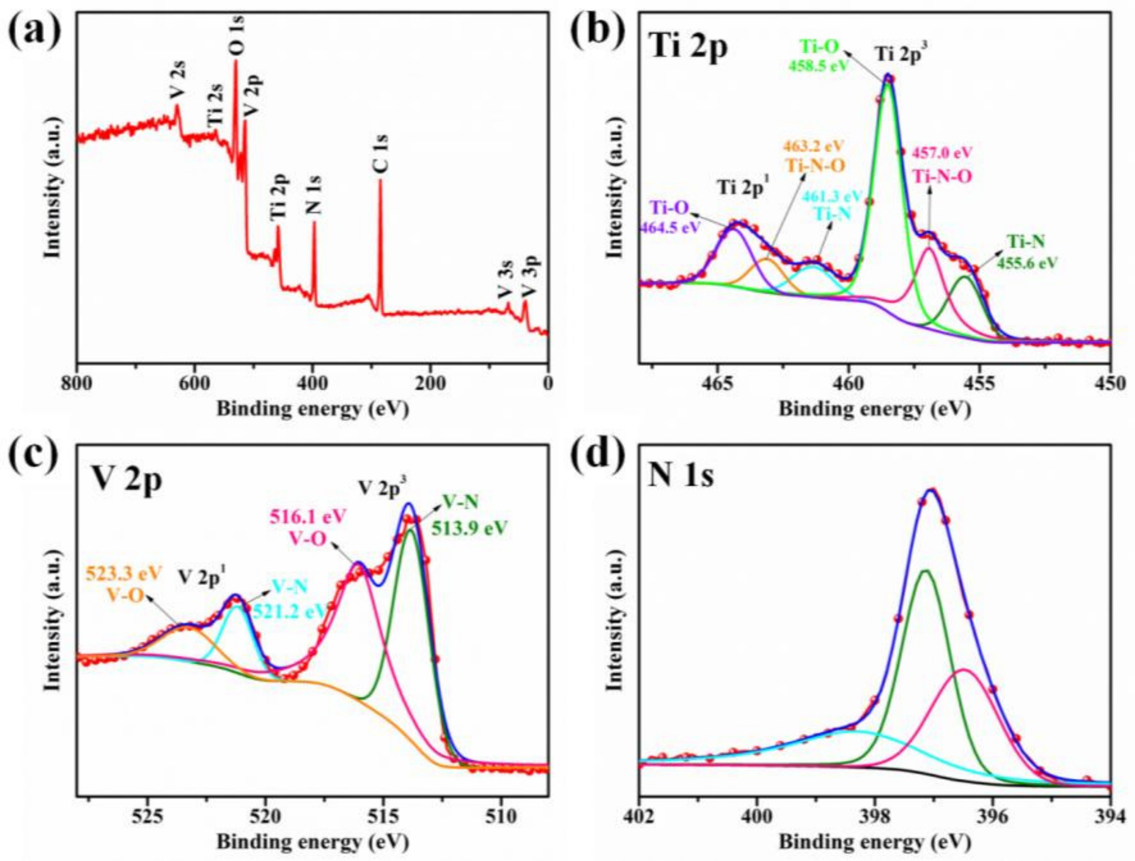

Figure S18 (a) XPS survey spectrum and (b-d) high-resolution spectra of Ti (b), V (c), N 1s (d), showed in the TiN NWAs@VN NSs/CNTF.

The survey spectrum in Figure S18 indicates that the TiN NWAs@VN NSs/CNTF consists of Ti, V, N, O, and C, in which the O comes from the oxygen containing group of C-O in the CNTF and the C derives from the CNTF and amorphous carbon derived from the annealing process. Figure S18(b)-(d) displays the typical Ti 2p, V 2p, and N 1s narrow scan of the TiN NWAs@VN NSs/CNTF. The Ti 2p can be divided into Ti $2p_{1/2}$ and Ti $2p_{3/2}$. The Ti $2p_{1/2}$ region shows three peaks at 464.5, 463.2 and 461.3 eV, and the Ti $2p_{3/2}$ region also exhibits three peaks at 458.5, 457.0 and 455.6 eV (Figure S18(b)). The peaks at 461.3 and 455.6 eV can be attributed to TiN. The peaks at 463.2 and 457.0 can be assigned to titanium oxynitride (Ti-N-O) while that at 464.5 and 458.5 eV can be attributed to Ti-O. Figure S18(c)

shows the high-resolution V 2p XPS spectra and V 2p can be can be divided into V $2p_{1/2}$ and V $2p_{3/2}$. The peaks at 521.2 and 513.9 eV are ascribed to the V $2p_{1/2}$ and V $2p_{3/2}$ signals of V-N. The peaks at 523.3 and 516.1 eV are attributed to V $2p_{1/2}$ and V $2p_{3/2}$ signals of V-O. In the high-resolution N1s XPS spectra (Figure S18(d)), binding energy at 396.4 eV corresponding to TiN and VN while that at 397.2 and 398.9 eV corresponding to Ti-N-O and V-O-N, respectively.

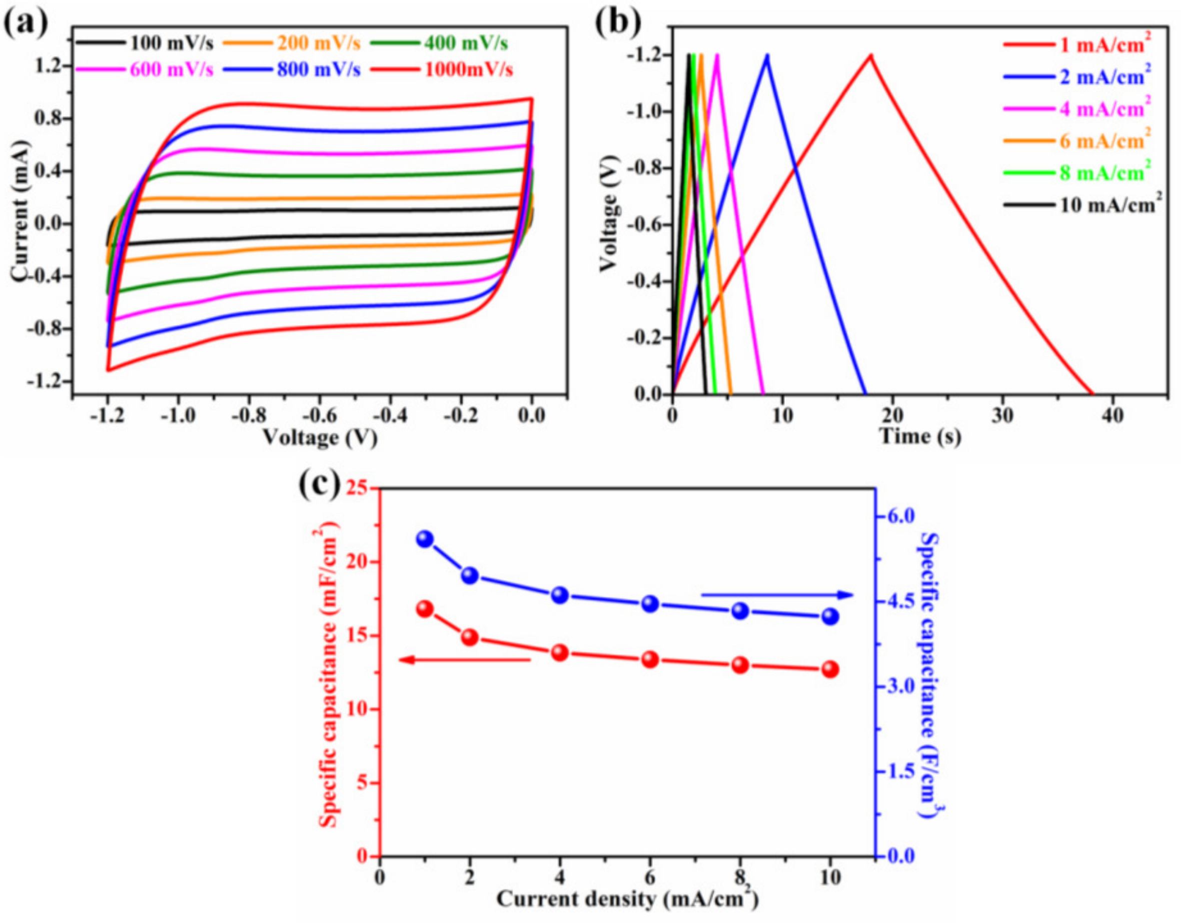

Figure S19 (a) CV curves of the bare CNTF electrode (used as negative electrode) in the potential window ranging from -1.2 to 0 V at various scan rates. (b) GCD curves of bare CNTF electrode in the potential window ranging from -1.2 to 0 V at different current densities. (c) Specific capacitances of the bare CNTF calculated from the corresponding discharge curves as a function of current density.

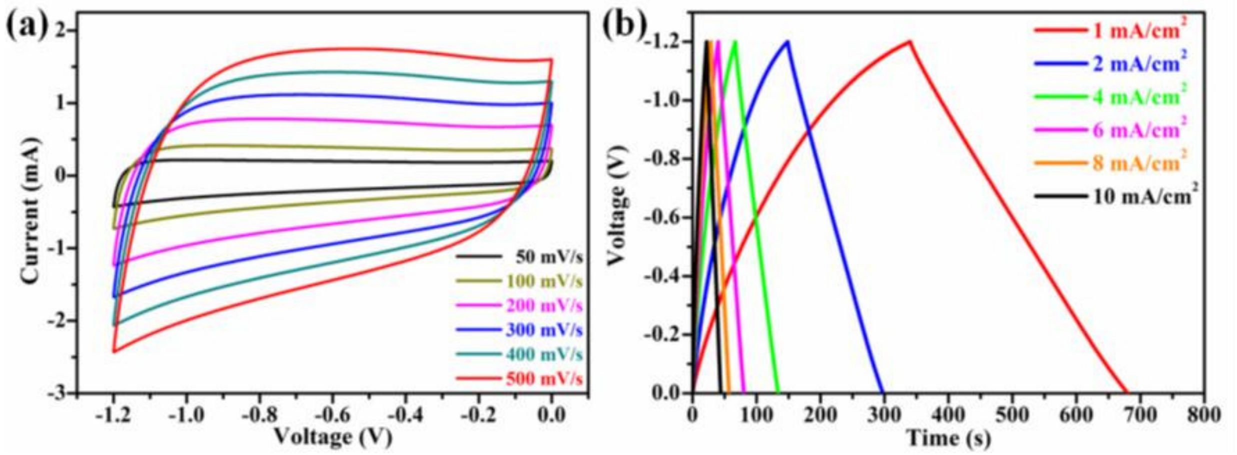

Figure S20 (a) CV curves of the TiN NWAs/CNTF electrode at different scan rates between -1.2 V-0 V. (b) GCD curves of the TiN NWAs/CNTF electrode at different current densities.

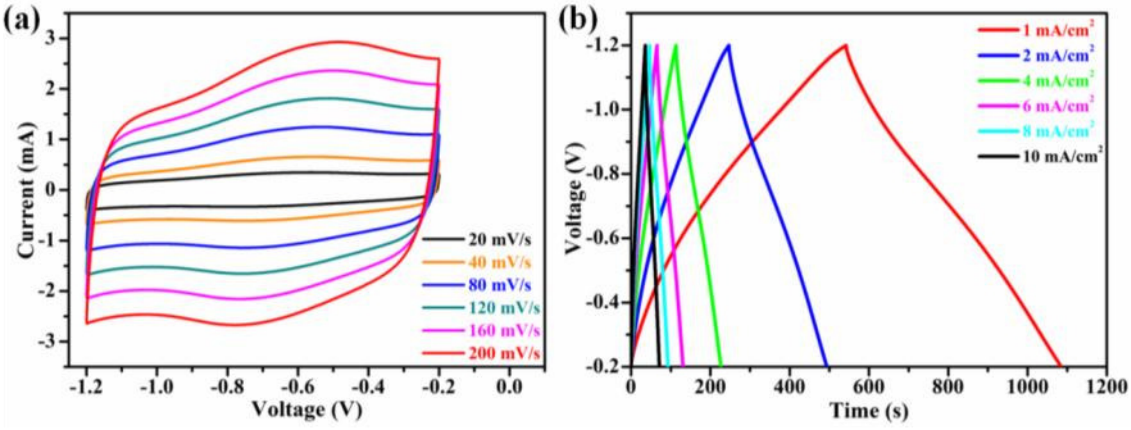

Figure S21 (a) CV curves of the VN NSs/CNTF electrode at different scan rates between -1.2 V-0.2 V. (b) GCD curves of the VN NSs/CNTF electrode at different current densities.

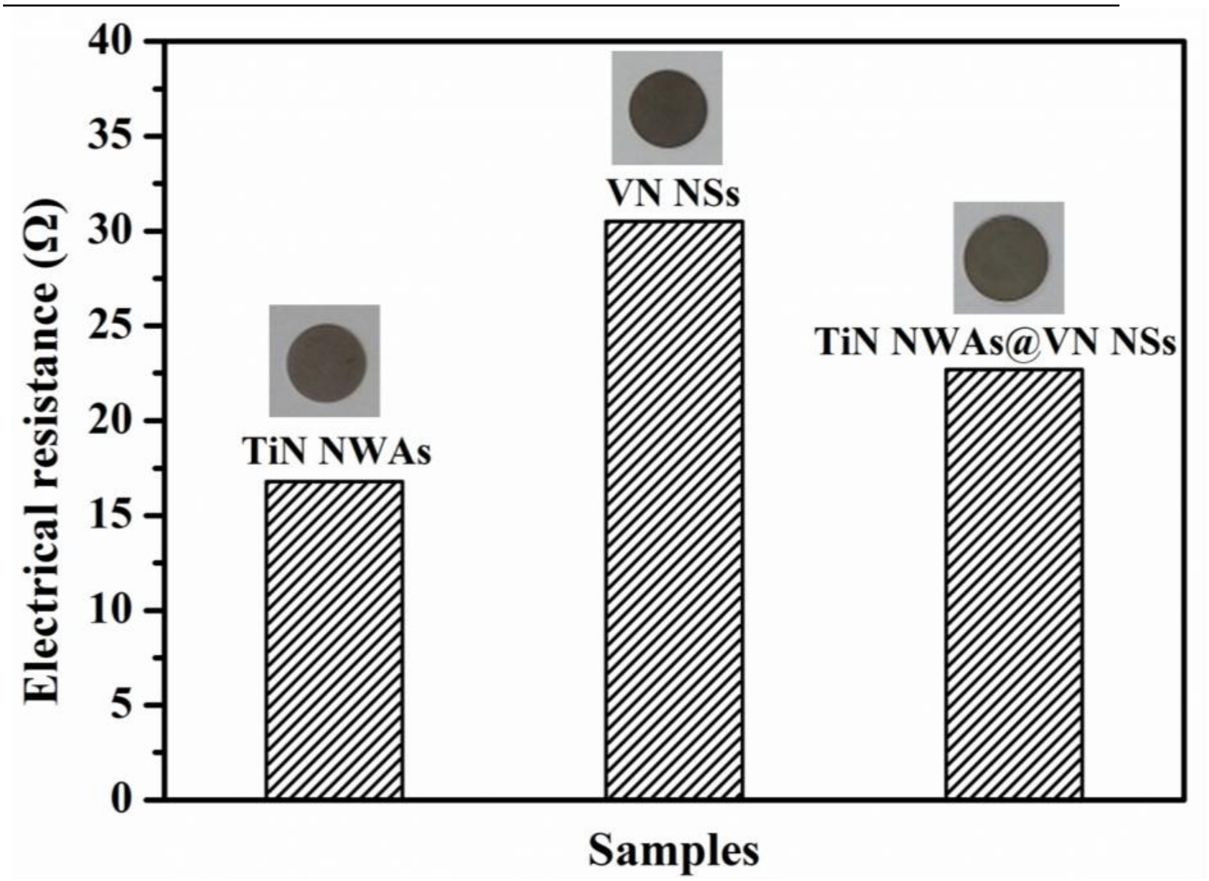

Figure S22 The compacted electrical resistance of the as-synthesized TiN NWAs, VN NSs and TiN NWAs@VN NSs powders.

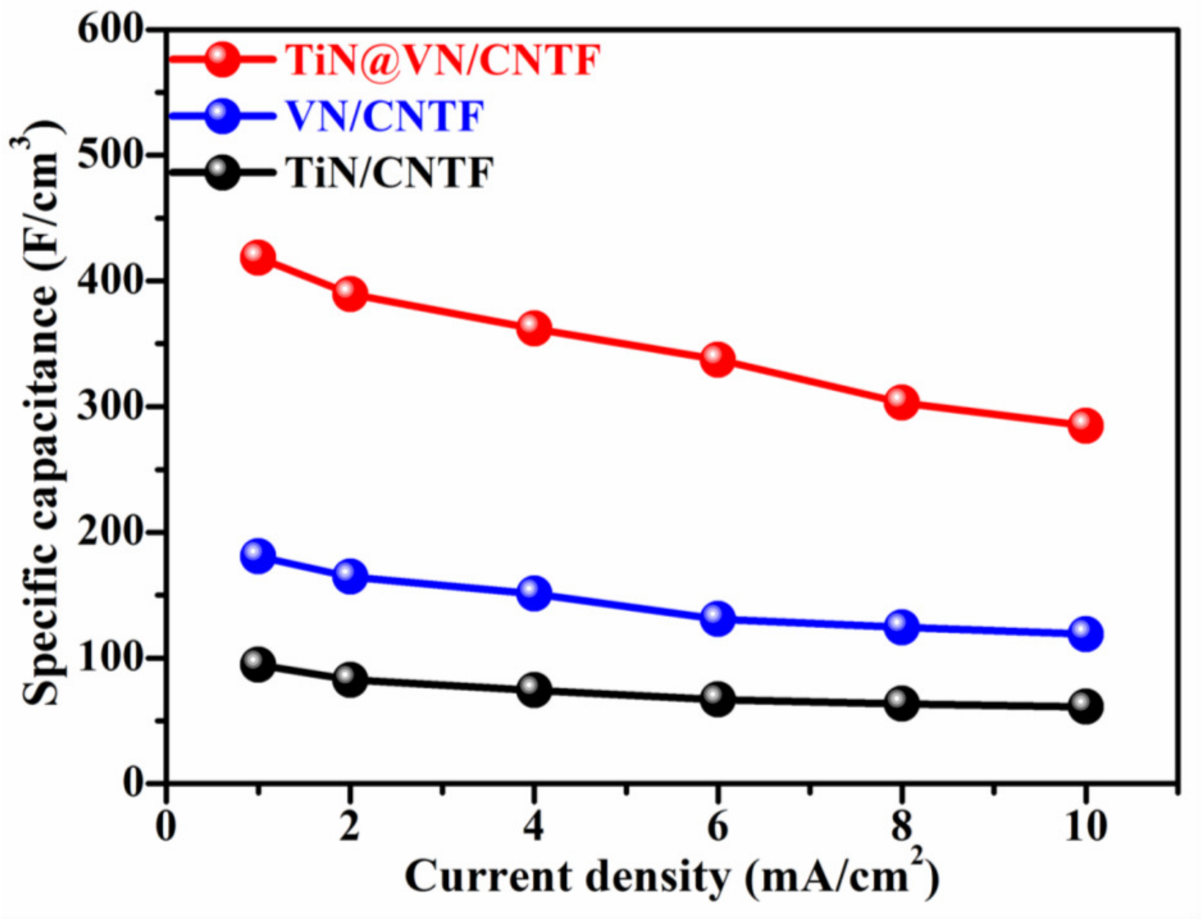

Figure S23 Specific capacitances of the TiN NWAs@VN NSs/CNTF, TiN NWAs/CNTF and VN NSs/CNTF electrodes as a function of the current density.

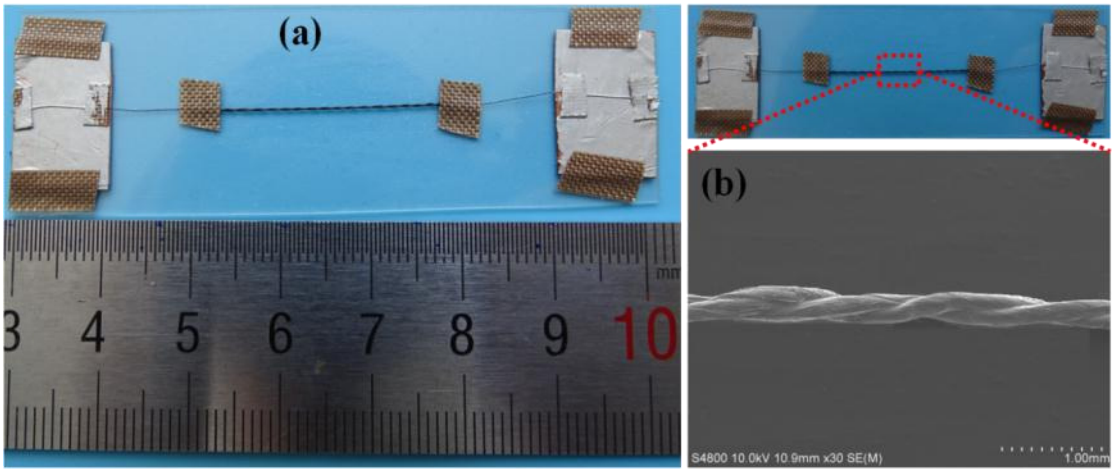

Figure S24 (a) Optical image and (b) SEM image of the as-assembled FASC device with twisted structure.

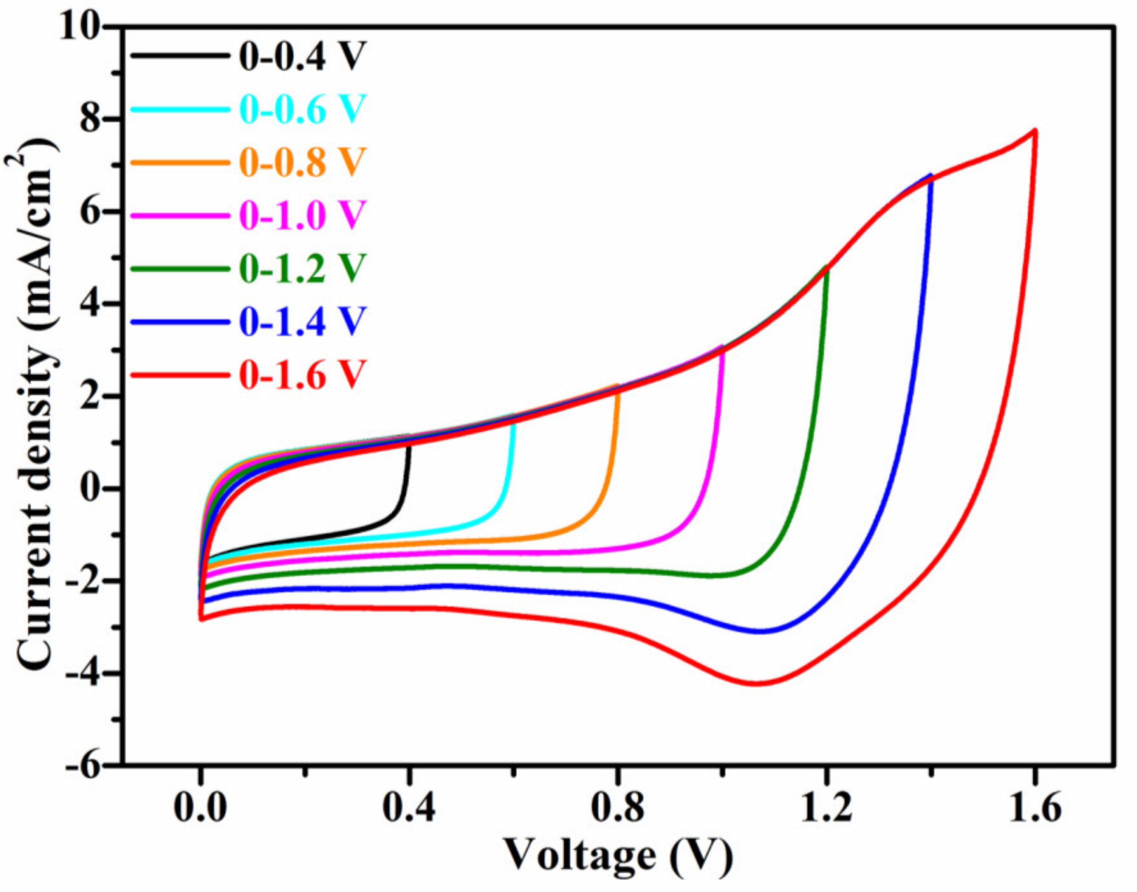

Figure S25 CV curves of the as-assembled FASC device measured at different operating voltages (0.4-1.6 V) at a constant scan rate of 25 mV/s.

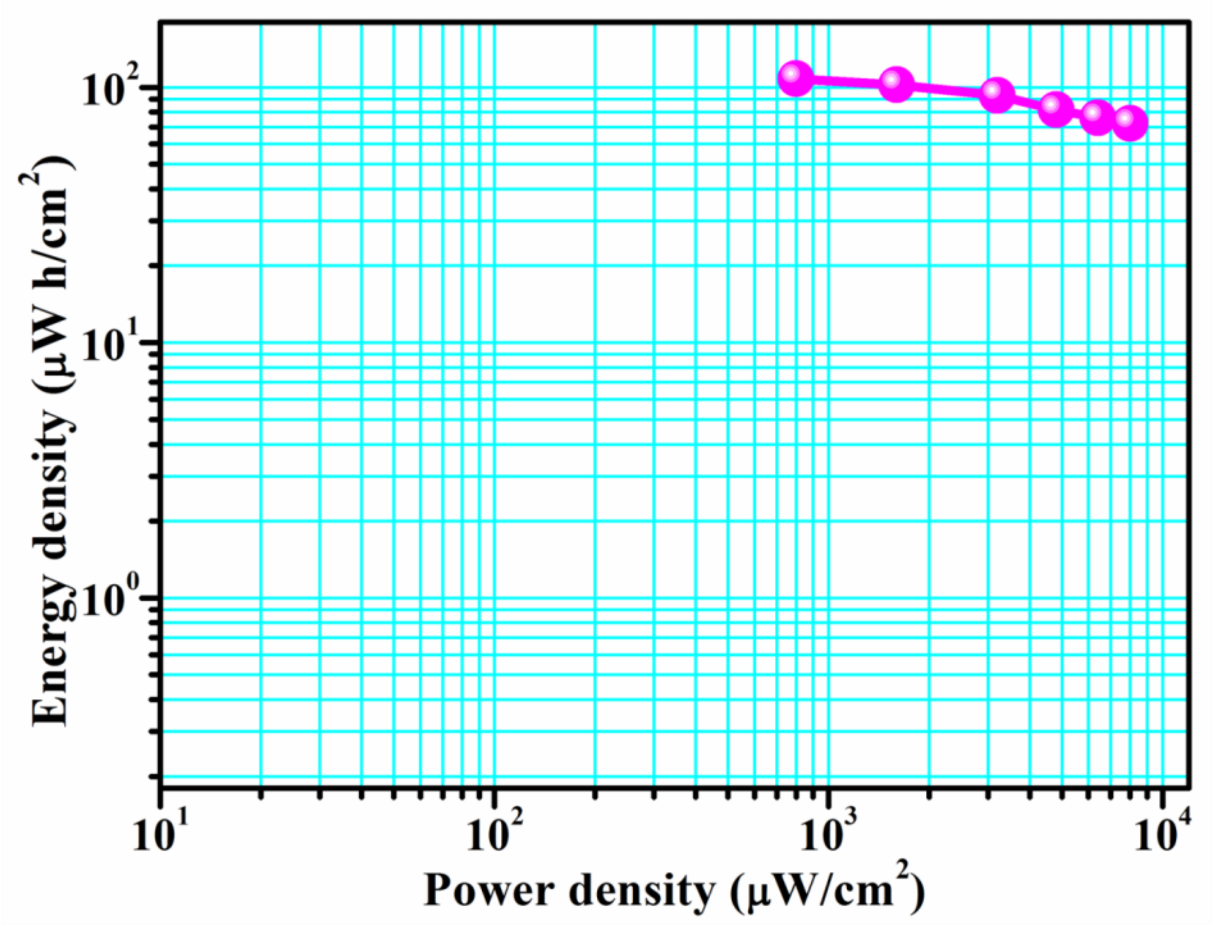

Figure S26 Areal energy and power densities of the as-assembled FASC device.

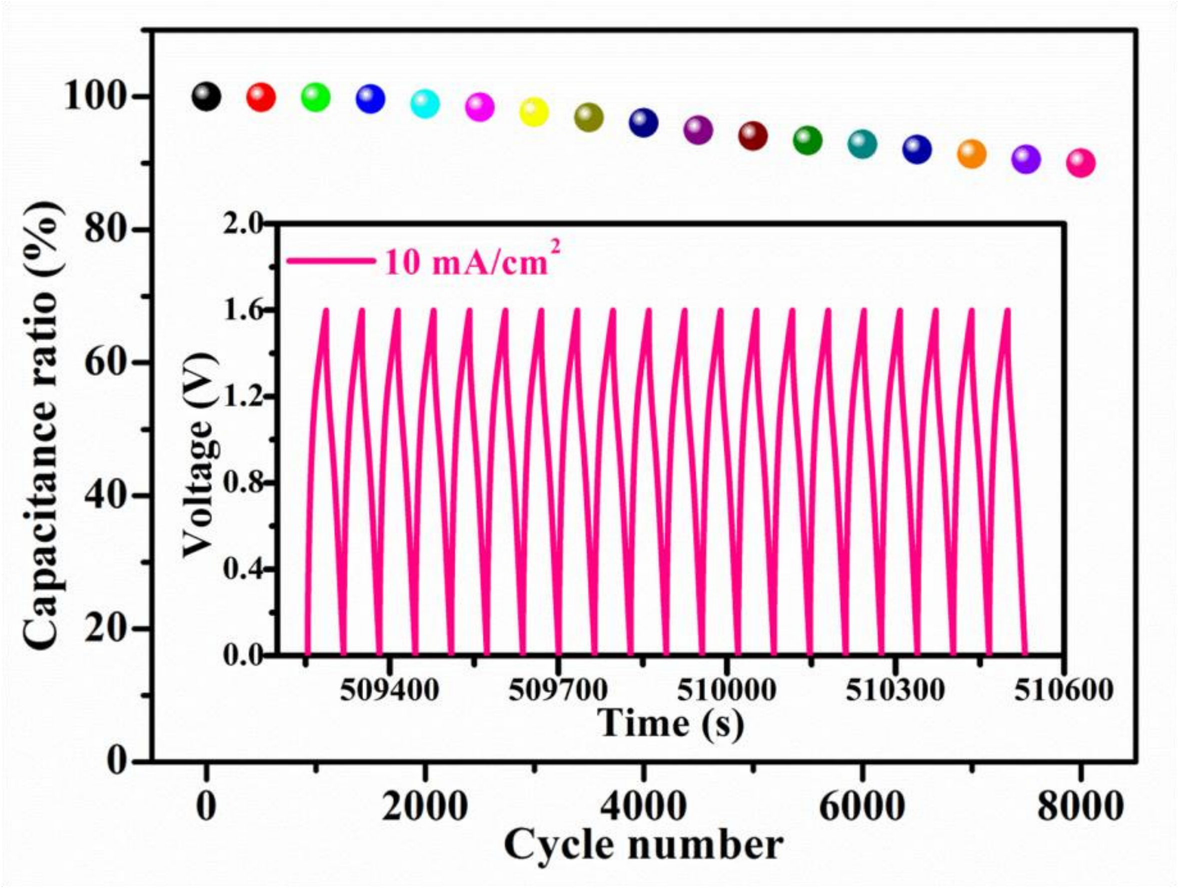

Figure S27 Cycling performance of the as-assembled FASC device at 10 mA/cm^2 (Inset is the GCD curves of the device for the last twenty cycles).

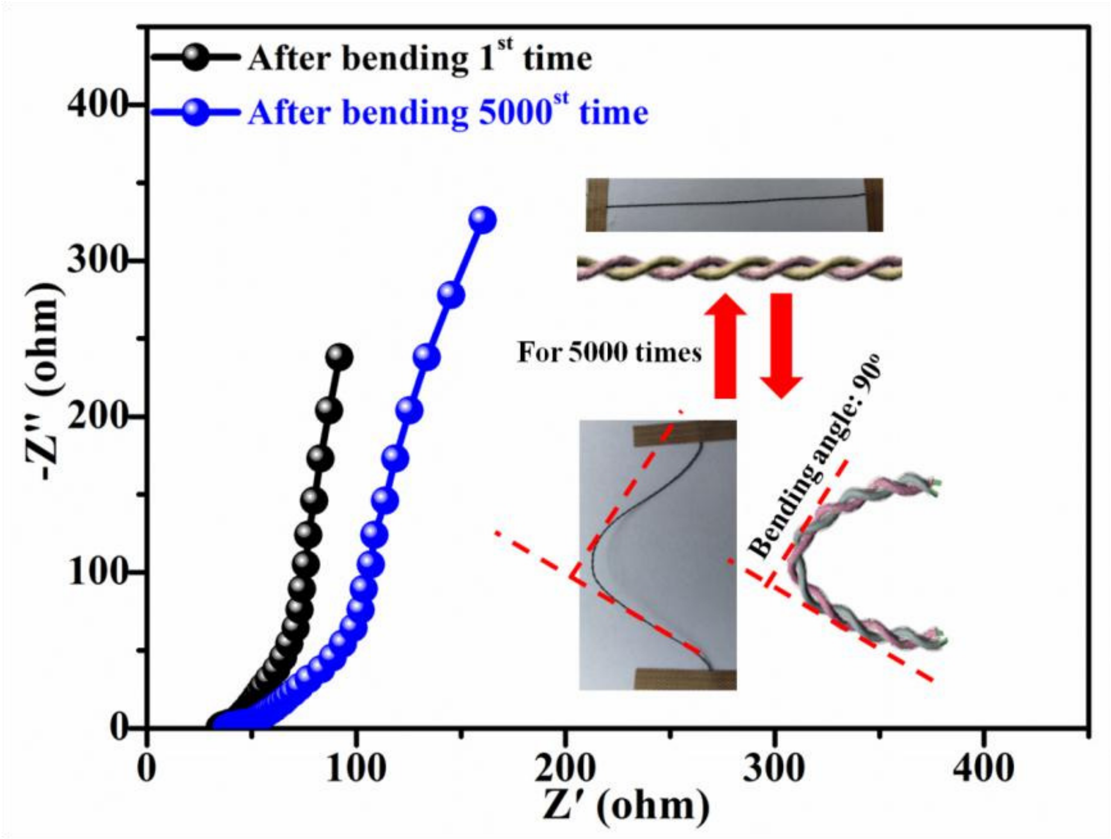

Figure S28 Nyquist plot of the as-assembled FASC device at frequencies ranging from 10^{-2} to 10^{5} Hz with a voltage amplitude of 5 mV at open-circuit potential after bending 1st and 5000th times (insert is the schematic diagram and pictures of the FASC device at a bending angle of 90° for 5000 cycles).

After the 1st and 5000th bending cycles, the equivalent series resistance values of our device are about 34.7 Ω and 38.2 Ω, indicating the improvements of the ionic resistance of the electrolyte and the resistance of the electrode material after the bending cycles. Moreover, the semicircle diameter related to charge transfer resistance also increases after 5000 bending cycles.

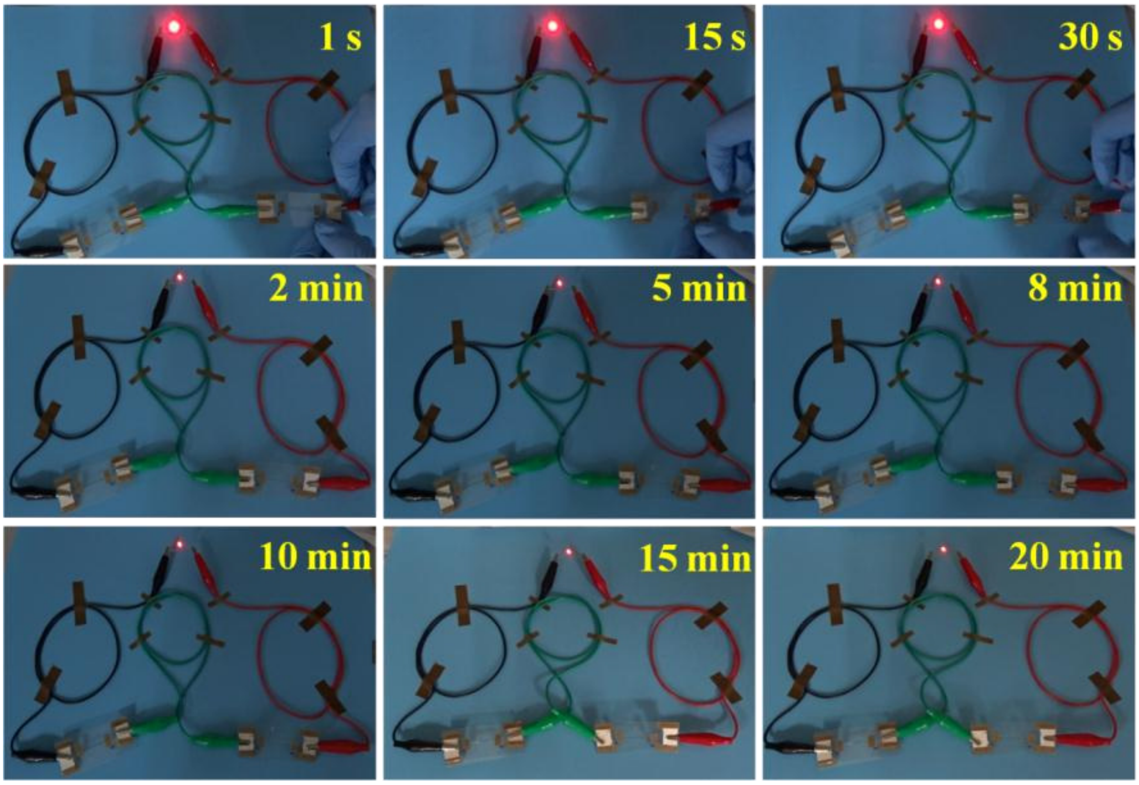

Figure S29 Optical images of lightness of a red LED (powered by two charged FASC device connected in series) changes with time.

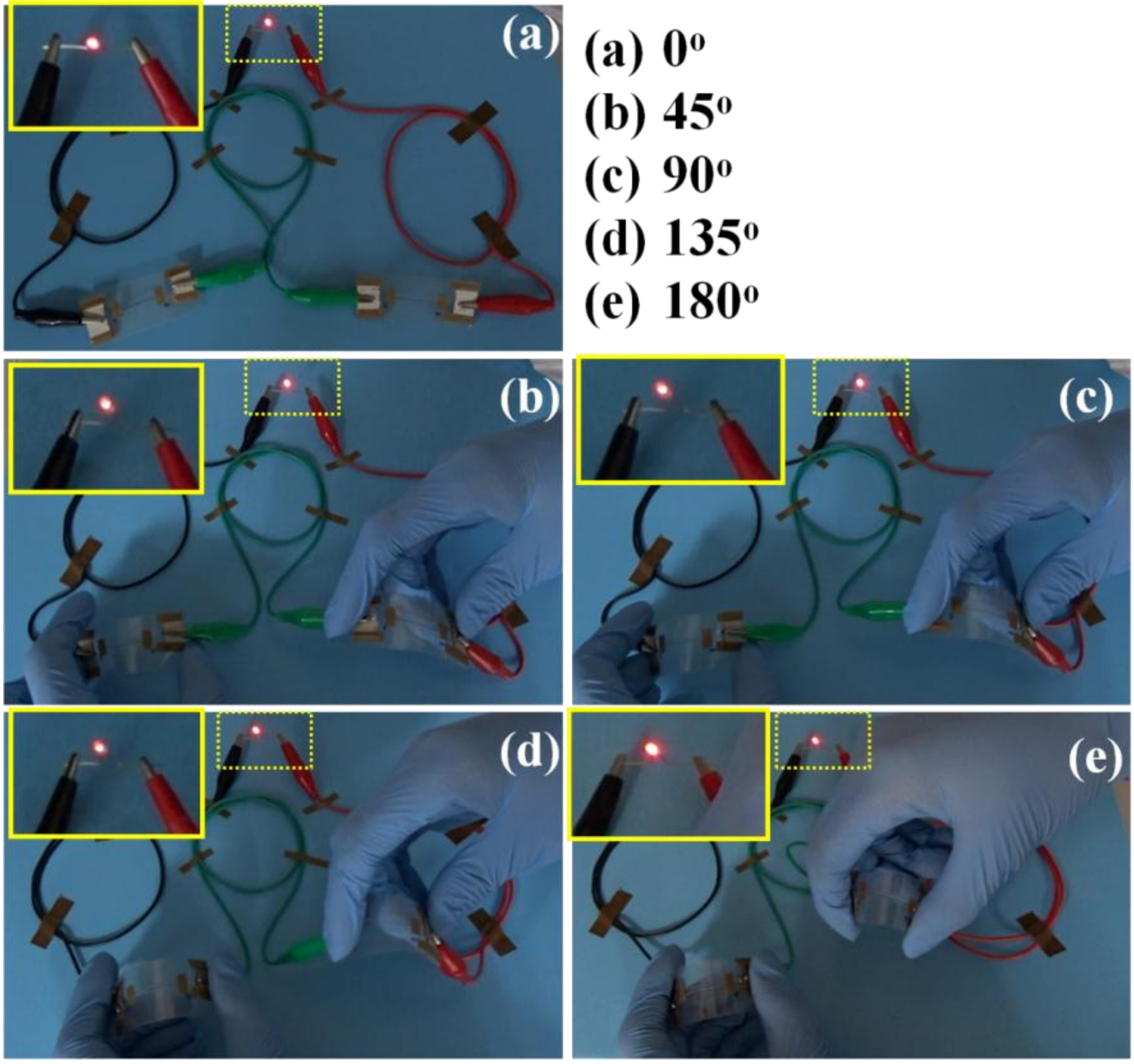

Figure S30 Optical images of the brightness of a red LED powered by two charged FASC device under different bending angle: (a) 0°, (b) 45°, (c) 90°, (d) 135°, and (e) 180°.

Table S1 Comparison of the specific capacitance, capacity and rate capability of our $CoNiO_2$ NWAs@$Ni(OH)_2$ NSs/CNTF electrode with previously reported positive electrodes.

Cathode material with core-shell structure	Area capacity ($\mu Ah/cm^2$)	Mass capacity (mAh/g)	Current density	Rate capability	Ref.
$MnCo_2O_4$@$Ni(OH)_2$ flowers	-	269	5 A/g	32.6% (20 A/g)	[1]
$NiCo_2O_4$@MnO_2 nanowire arrays	551.7	-	2 mA/cm^2	50.2% (20 mA/cm^2)	[2]
CoO@PPy nanowire arrays	553.8	-	1 mA/cm^2	28.9% (50 mA/cm^2)	[3]
Co_3O_4@NiO nanowire arrays	187.5	69.1	6 mA/cm^2 (2 A/g)	85.0% (40 A/g)	[4]
$NiCo_2O_4$@$MnMoO_4$ nanocolumn arrays	265.3	-	1 mA/cm^2	62.3% (20 mA/cm^2)	[5]
MnO_2@NiO tubular array	66.7	-	5 mA/cm^2	55.0% (25 mA/cm^2)	[6]
Ni_3S_2 nanorod@$Ni(OH)_2$ nanosheet	588.2	158.5	19 .1 mA/cm^2 (5.1 A/g)	38.4% (19.8 A/g)	[7]
$CoMoO_4$@$CoNiO_2$ nanowire arrays	885	-	5 mA/cm^2	33.0% (100mA/cm^2)	[8]
H-CoOx nanowire@$Ni(OH)_2$ nanosheet	820.4	335.5	5.2 mA/cm^2 (2 A/g)	67.9% (50 A/g)	[9]
$NiMoO_4$@$Co(OH)_2$ nanowire arrays	324.3	294.8	5 mA/cm^2 (4.5 A/g)	38.9% (50 mA/cm^2)	[10]
Co_9S_8 nanorod@$Ni(OH)_2$ nanosheet	-	248	0.5 A/g	75.8% (20 A/g)	[11]
Co_3O_4@$Ni(OH)_2$ nanosheet arrays		199.6	1.2 A/g	45.9% (12.1 A/g)	[12]
$NiCo_2S_4$/$Ni(OH)_2$ nanotube arrays	-	338	1 mA/cm^2	59.3% (20 mA/cm^2)	[13]
TiO_2@$Ni(OH)_2$ nanowire arrays	-	264	1 A/g	67.4% (10 A/g)	[14]
$CoNiO_2$ nanowires	-	132.6	2 A/g	44.8% (50 mA/cm^2)	[15]
**$CoNiO_2$ NWAs@$Ni(OH)_2$ NSs/CNTF**	**674.1**	**539.3**	**1 mA/cm^2 (0.8A/g)**	**69.8% (10 mA/cm^2)**	**This work**

Table S2. Comparison of the specific capacitance and rate capability of the TiN NWAs@VN NSs/CNTF electrode with previously reported negative electrodes.

Anode materials	Specific capacitance (mF/cm^2)	Specific capacitance (F/cm^3)	Specific capacitance (F/g)	Current density	Rate performance	Ref.
TiN@V_2O_5 NWAs/CNTF	692	230.7	-	2 mA/cm^2	51.3% (20 mA/cm^2)	[16]
MoNx	52.45	-	174.83	1.5 A/g	62.4% (30 A/g)	[17]
Flower-like MoS_2 nanosheets/CNTF	1076	-	-	1 mA/cm^2	67.2% (10 mA/cm^2)	[18]
VN nanosheets/CNTF	564	188	-	1 mA/cm^2	64.0% (10 mA/cm^2)	[19]
MoNx/TiN nanotube array	121.5	-	-	0.3 mA/cm^2	59.9% (6 mA/cm^2)	[17]
TiN@C/carbon cloth			159.0	0.25 A/g	78.3% (5 A/g)	[20]
TiN	-	-	407	1 A/g	76.7% (10 A/g)	[21]
VN quantum dot/N-doped microporous CNF	-	-	406.5	0.5 A/g	75.1% (5 A/g)	[22]
TiVN thin film	-	-	67	0.1 mA/g	73.2% (1 mA/g)	[23]
graphene/TiN nanotube array	-	-	333.7	1 A/g	53.3% (10 A/g)	[24]
VN@C NWAs/CNTF	715	-	-	1 mA/cm^2	68.8% (10 mA/cm^2)	[25]
VN/N-doped graphene	-	-	445	1 A/g	62.5% (50 A/g)	[26]
**TiN NWAs@VN NSs/CNTF**	**1255.2**	**418.4**	**1380.7**	**1 mA/cm^2 (1.1 A/g)**	**68.1% (10 mA/cm^2)**	**This work**

References

[1] Y. Zhao, L. Hu, S. Zhao, L. Wu, Adv. Funct. Mater. **2016**, 26, 4085.

[2] L. Yu, G. Zhang, C. Yuan, X. Lou, Chem. Commun. **2013**, 49, 137.

[3] C. Zhou, Y. Zhang, Y. Li, J. Liu, Nano Lett. **2013**, 13, 2078.

[4] X. Xia, J. Tu, Y. Zhang, X.Wang, C. Gu, X. Zhao, H. Fan, ACS Nano, **2012**, 6, 5531.

[5] C. Cui, J. Xu, L. Wang, D. Guo, M. Mao, J. Ma, T. Wang, ACS Appl. Mater. Interfaces, **2016**, 8, 8568.

[6] J. Liu, J. Jiang, M. Bosman, H. Fan, J. Mater. Chem. **2012**, 22, 2419.

[7] W. Zhou, X. Cao, Z. Zeng, W. Shi, Y. Zhu, Q. Yan, H. Liu, J. Wang, H. Zhang, Energy Environ. Sci. **2013**, 6, 2216.

[8] Y. Ai, X. Geng, Z. Lou, Z. Wang, G. Shen, ACS Appl. Mater. Interfaces **2015**, 7, 24204.

[9] J. Zhu, L. Huang, Y. Xiao, L. Shen, Q. Chen, W. Shi, Nanoscale **2014**, 6, 6772.

[10] W. Ren, D. Guo, M. Zhuo, B. Guan, D. Zhang, Q. Li, RSC Adv. **2015**, 5, 21881.

[11] J. Wen, S. Li, B. Li, Z. Song, H. Wang, R. Xiong, G. Fang, J. Power Sources **2015**, 284, 279.

[12] X. Bai, Q. Liu, J. Liu, H. Zhang, Z. Li, X. Jing, P. Liu, J. Wang, R. Li, Chem. Eng. J. **2017**, 315, 35.

[13] J. Zhang, H. Gao, M. Zhang, Q. Yang, H. Chuo, Appl. Surf. Sci. **2015**, 349, 870.

[14] Ke Q, M. Zheng, H. Liu, C. Guan, L. Mao, J. Wang, Sci. Rep. **2015**, 5, 13940.

[15] Y. Ai, Z. Lou, L. Li, S. Chen, H.S. Park, Z.M. Wang, G. Shen, Adv. Mater. Technol. **2016**, 1, 1600142.

[16] J. Guo, Q. Zhang, Q. Li, J. Sun, C. Li, B. He, Z. Zhou, L. Xie, M. Li, Y. Yao, ACS Appl. Mater. Interfaces **2018**, 10, 29705.

[17] Y. Xie, F. Tian, Mater. Sci. Eng. B **2017**, 215, 64.

[18] Q. Zhang, J. Sun, Z. Pan, J. Zhang, J. Zhao, X. Wang, C. Zhang, Y. Yao, W. Lu, Q. Li, Y. Zhang, Z. Zhang, Nano Energy **2017**, 39, 219.

[19] J. Guo, Q. Zhang, J. Sun, C. Li, J. Zhao, Z. Zhou, B. He, X. Wang, P. Man, Q. Li, J. Zhang, L. Xie, M. Li, Y. Yao, J. Power Sources **2018**, 382, 122.

[20] X. Lu, T. Liu, T. Zhai, G. Wang, M. Yu, S. Xie, Y. Ling, C. Liang, Y. Tong, Y. Li, Adv. Energy Mater. **2014**, 4, 1300994.

[21] S. Tang, Q. Cheng, J. Zhao, J. Liang, C. Liu, Q. Lan, Y.-C. Cao, J. Liu, Results Phys. **2017**, 7, 1198.

[22] Y. Wu, F. Ran, J. Power Sources **2017**, 344, 1.

[23] V.V. Anusha Thampi, U. Nithiyanantham, A.K. Nanda Kumar, P. Martin, A. Bendavid, B. Subramanian, J. Mater. Sci. Mater. El. **2018**, 29, 12457.

[24] F. Tian, Y. Xie, H. Du, Y. Zhou, C. Xia, W. Wang, RSC Adv. **2014**, 4, 41856.

[25] Q. Zhang, X. Wang, Z. Pan, J. Sun, J. Zhao, J. Zhang, C. Zhang, L. Tang, J. Luo, B. Song, Z. Zhang, W. Lu, Q. Li, Y. Zhang, Y. Yao, Nano Lett. **2017**, 17, 2719.

[26] J. Balamurugan, G. Karthikeyan, T. Thanh, N. Kim, J. Lee, J. Power Sources **2016**, 308, 149.
